# DAXX-ATRX regulation of p53 chromatin binding and DNA damage response

**DOI:** 10.1038/s41467-022-32680-8

**Published:** 2022-08-26

**Authors:** Nitish Gulve, Chenhe Su, Zhong Deng, Samantha S. Soldan, Olga Vladimirova, Jayamanna Wickramasinghe, Hongwu Zheng, Andrew V. Kossenkov, Paul. M. Lieberman

**Affiliations:** 1 The Wistar Institute, Philadelphia, PA 19104 USA; 2 Weill School of Medicine, Cornell University, New York, NY USA

**Keywords:** Tumour-suppressor proteins, Telomeres

## Abstract

DAXX and ATRX are tumor suppressor proteins that form a histone H3.3 chaperone complex and are frequently mutated in cancers with the alternative lengthening of telomeres (ALT). Here, we show that DAXX and ATRX knock-out (KO) U87-T cells that have acquired ALT-like features have defects in p53 chromatin binding and DNA damage response. RNA-seq analysis revealed that p53 pathway is among the most perturbed. ChIP-seq and ATAC-seq revealed a genome-wide reduction in p53 DNA-binding and corresponding loss of chromatin accessibility at many p53 response elements across the genome. Both DAXX and ATRX null cells showed a depletion of histone H3.3 and accumulation of γH2AX at many p53 sites, including subtelomeres. These findings indicate that loss of DAXX or ATRX can compromise p53 chromatin binding and p53 DNA damage response in ALT-like cells, providing a link between histone composition, chromatin accessibility and tumor suppressor function of p53.

## Abstract (web summary)

The tumor suppressor proteins DAXX and ATRX are frequently mutated in cancers with alternative lengthening of telomeres (ALT). This study shows that DAXX-ATRX regulates p53 chromatin accessibility and DNA damage response and that disruption of this pathway is critical for ALT cell survival.

## Introduction

DAXX and ATRX have been implicated as tumor-suppressor proteins that restrict the alternative lengthening of telomeres (ALT)^1–4^. ALT is a recombination-based mechanism that arises in 10–15% of cancer through poorly understood genetic and epigenetic changes^5–8^. Whole genome sequencing studies have identified loss of function mutations in ATRX, DAXX, and histone H3.3 to be significantly associated with ALT induction in different classes of gliomas^9–11^, sarcomas^12,13^, and pancreatic neuroendocrine (PanNET) tumors^4,14–16^. Moreover, analysis of 22 immortalized ALT cell lines underscored the involvement of the ATRX/DAXX complex in ALT activation^17^. ALT cells modulate chromatin and DNA repair pathways that facilitate telomere elongation through homologous DNA recombination^10,18^. Telomere recombination is known to be inhibited by telomere repeat binding factors and telomeric heterochromatin^19,20^. Both DAXX or ATRX are known to contribute to telomeric heterochromatin and to limit homologous recombination^1–3,9^. However, loss of DAXX or ATRX is not sufficient to establish telomere recombination and cell proliferation associated with ALT^10,11^.

DAXX and ATRX are thought to suppress ALT, in part, through their ability to load histone H3.3 into chromatin^1,10,21^. ALT cells have more accessible telomeric heterochromatin and accumulate high levels of single-strand DNA and DNA damage marked by association with γH2AX and 53BP1 at telomeres^10^. This DNA-damage signaling reflects both double-strand breaks and homologous recombination repair. The extent and tolerance of DNA damage increases further upon mutation of TP53 (p53)^13^. TCGA pan-cancer atlas study of ALT-related genes shows *TP53* to be one of the top mutated genes^14^. p53 is a DNA-binding protein and master transcriptional regulator of genome integrity controlling transcription of gene networks for cellular senescence and apoptosis^15,16^. p53 binds to several loci within the cellular genome, including promoters, enhancers, transcriptionally inactive regions as well as retrotransposon-like elements in many human subtelomeres^17,18^. The inter-relationship between DAXX-ATRX, and p53 DNA-damage response function in ALT is not completely understood.

Both DAXX and ATRX are multifunctional tumor-suppressor proteins^1,4^. ATRX and DAXX form a complex together with the histone variant H3.3 which is responsible for depositing H3.3 at heterochromatic regions of the genome, including telomeres^22–24^. In addition to their function in loading H3.3 onto telomeric chromatin, DAXX and ATRX bind many other proteins, including p53 and MDM2, and can localize with PML-containing nuclear bodies (PML-NBs) that associate with ALT telomeres. ATRX/DAXX/H3.3 and p53 mutations frequently co-occur in ALT cancers^9^, suggesting that these pathways may be additive in cancer cell progression. In a previous study, we demonstrated that loss of ATRX or DAXX is not sufficient for ALT, but that additional genetic and/or epigenetic events can lead to the emergence of clones with ALT-like features^10^. Here, we investigate the relationship between DAXX and ATRX loss of function and p53 DNA-damage sensing pathway using sets of syngenic glioblastoma cell lines with CRISPR/Cas9 knockout of DAXX or ATRX that have acquired ALT-like features relative to parental controls. Our findings suggest that the DAXX-ATRX regulates p53 chromatin accessibility and DNA-damage response, that H3.3 may also be involved in this mechanism, and that disruption of this pathway is critical for ALT cell survival.

## Results

### Deregulation of p53 pathway in DAXX and ATRX-knockout cells with ALT-like features

**Fig. 1:**
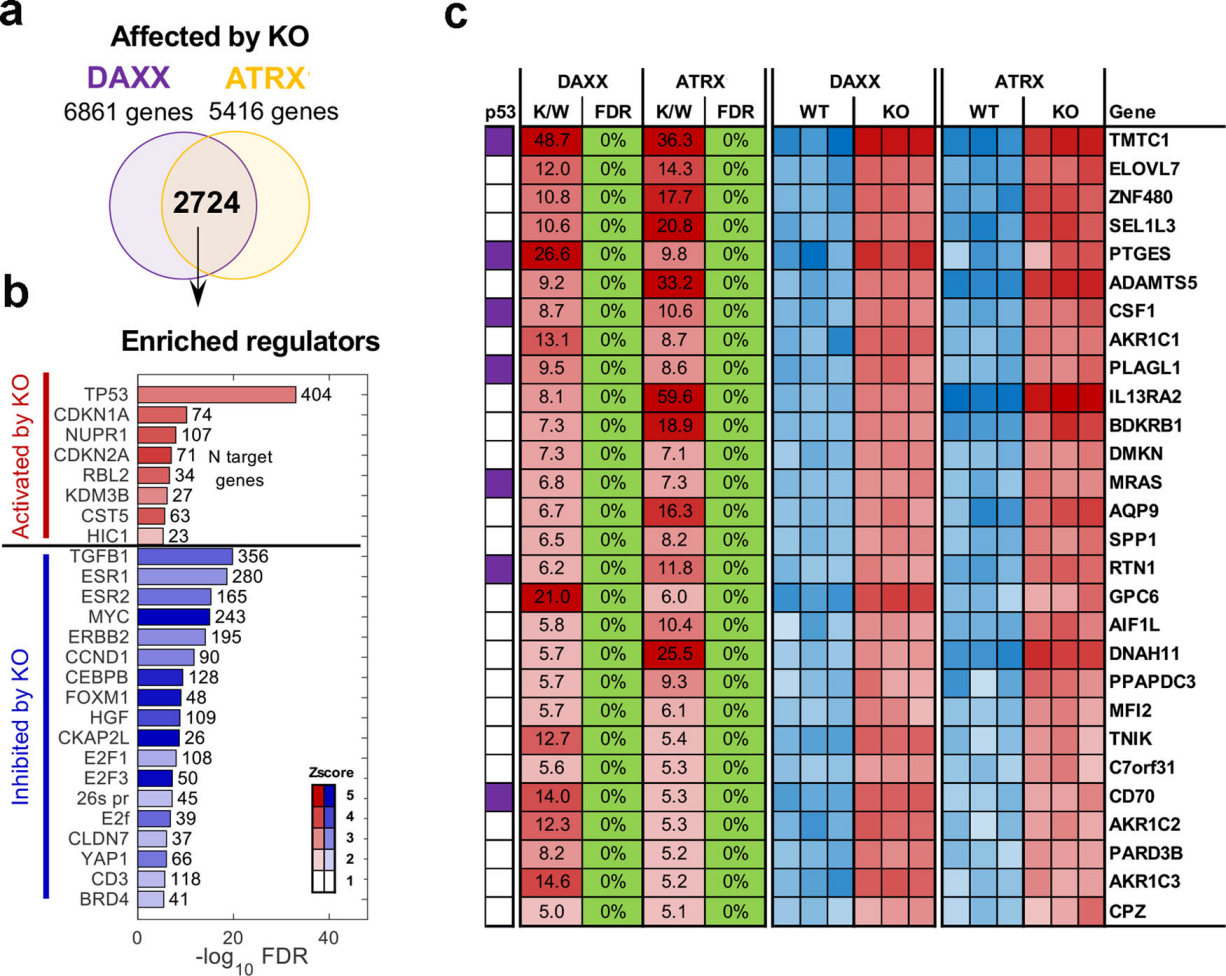
Transcriptomic profile of DAXX_KO and ATRX_KO U87 cells. **a** Venn diagram showing overlap of gene transcripts affected by DAXX_KO or ATRX_KO relative to control U87-T cells based on RNA-seq (Quantseq). **b** Significantly enriched regulators (FDR < 10^−5^, |Z| > 2) predicted by IPA to be activated (red) or inhibited (blue) by DAXX_KO and ATRX_KO. **c** Expression heatmap showing top differentially regulated genes (FDR < 5%, at least fivefold) for DAXX_KO and ATRX_KO relative to WT control. Ratio of KO to WT (K/W) and false discovery rate (FDR) are shown. Known direct p53 targets are indicated (purple).

We have previously reported the generation of ATRX and DAXX deficient U87-T glioblastoma cell lines that have acquired ALT-like features^10^. In that study, we found that transient depletion of DAXX or ATRX led to an increase in senescent cells, but that continuous passage enabled the isolation of clonal survivors with hallmarks of ALT. To gain further insight into the common features of these ALT-like DAXX_KO and ATRX_KO cells, we performed RNA-seq transcriptomic analysis. We found overlap of 2724 genes significantly (FDR < 5%) affected by both DAXX_KO and ATRX_KO relative to parental U87-T control cells (Fig. 1a). Gene enrichment analysis of the overlap revealed a number of significantly affected regulators (FDR < 10^−5^, Z > 2) with p53 reaching the most significance and predicted to be more active in the KO cell lines (Fig. 1b). Seven known p53 direct targets among 28 top upregulated genes (FDR < 5% in DAXX_KO and ATRX_KO, > fivefold upregulation) were identified, including TMTC1, PTGES, CSF1, PLAGL1, MRAS, RTN1, and CD70 (Fig. 1c). Several of these genes have been implicated in glioblastoma tumorigenesis^22–26^. An analysis of TCGA GBM dataset showed that the TP53 pathway is significantly downregulated in ATRX mutated GBM (Supplementary Fig. 1). The analysis also revealed that all of the ATRX mutated GBMs in TCGA had a corresponding loss of function mutations in TP53, consistent with other reports^27,28^, and suggesting these two pathways are mechanistically linked during GBM oncogenesis.

### Attenuated DNA-damage response in ALT-like cells lacking ATRX or DAXX

**Fig. 2:**
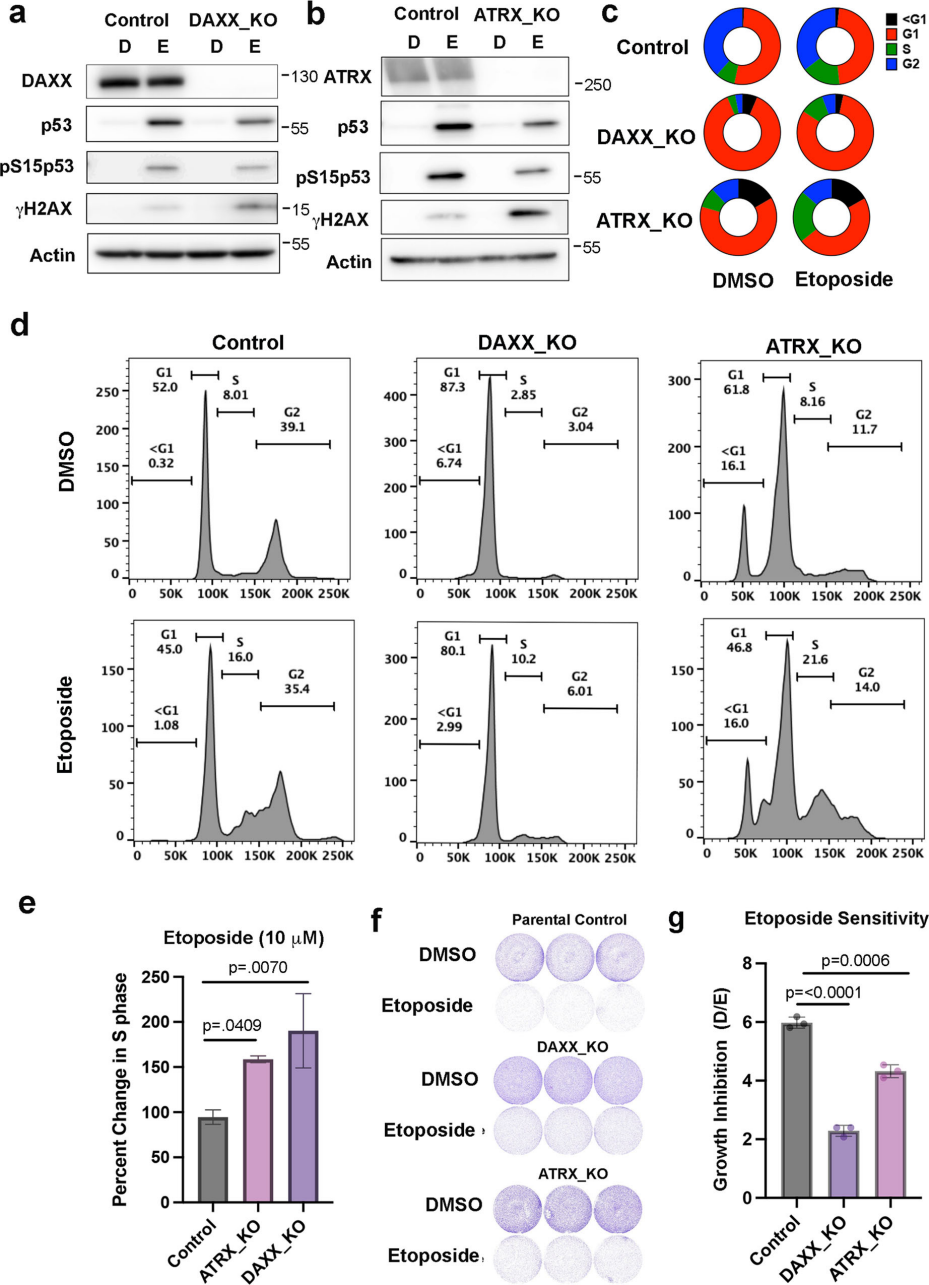
Aberrant DNA-damage response in DAXX_KO and ATRX_KO U87 cells. **a** Western blot for control or DAXX_KO cells treated with DMSO (D) or 50 μM etoposide (E) for 24 h probed with antibody to DAXX, p53, pS15p53, or actin. **b** Western blot for ATRX_KO cells treated same as in panel **a**. **c** Cell cycle distribution of U87-T Control, DAXX_KO, and ATRX_KO cells treated with DMSO or etoposide (10 µM) for 24 h (<G1 = black, G1 = red, S = green, G2 = blue). **d** FACS cell cycle profiles of propidium iodide-stained U87-T control, DAXX_KO, or ATRX_KO cells. **e** Percent increase in S phase populations in Etoposide (10 µM) treated cells compared to DMSO treated controls. (ANOVA *P* = 0.0079; Tukey’s multiple comparisons test *P* values indicated). **f** Clonogenicity assay with U87-T parental control, DAXX_KO, or ATRX_KO cells treated with DMSO or 50 μM etoposide followed by replating and staining with crystal violet. **g** Quantification of clonogenicity assay shown in panel **f** Error bars are sdm and *P* values determined by student *t* test for three biological replicates.

Given the transcriptomic alteration of p53-response pathway in ATRX_KO and DAXX_KO cells, we assessed their ability to respond to DNA damage relative to parental control U87-T cell line. We found that the topoisomerase inhibitor etoposide induced a potent p53 response and corresponding growth inhibition in parental U87-T cells (Fig. 2). Western blot analysis indicated that p53 and phospho-S15 p53 levels were induced in U87-T parental, but to a lesser extent in DAXX_KO (Fig. 2a) and ATRX_KO (Fig. 2b) cell lines. In contrast, γH2AX levels were modestly increased in KO cells relative to parental control. These findings suggest that KO cell lines have attenuated p53 response and accumulate higher levels of γH2AX DNA damage in response to etoposide treatment. Cell cycle distribution of each cell type with or without etoposide treatment was assayed by flow cytometry (Fig. 2c–e). We found that DAXX_KO and ATRX_KO cells were enriched in G1 and depleted in S/G2 relative to control under normal growth conditions, while the percentage in the S phase increased in KO cells relative to control after etoposide treatment. A clonogenicity assay also revealed that DAXX_KO and ATRX_KO had defects in growth inhibition relative to parental control in response to etoposide treatment (Fig. 2f, g). Taken together, these findings indicate that DAXX and ATRX KO U87-T cells have changes in cell cycle distribution and defects in cell cycle arrest in response to DNA damage.

### Altered p53-response transcriptome in U87 ALT-like cells lacking ATRX or DAXX

**Fig. 3:**
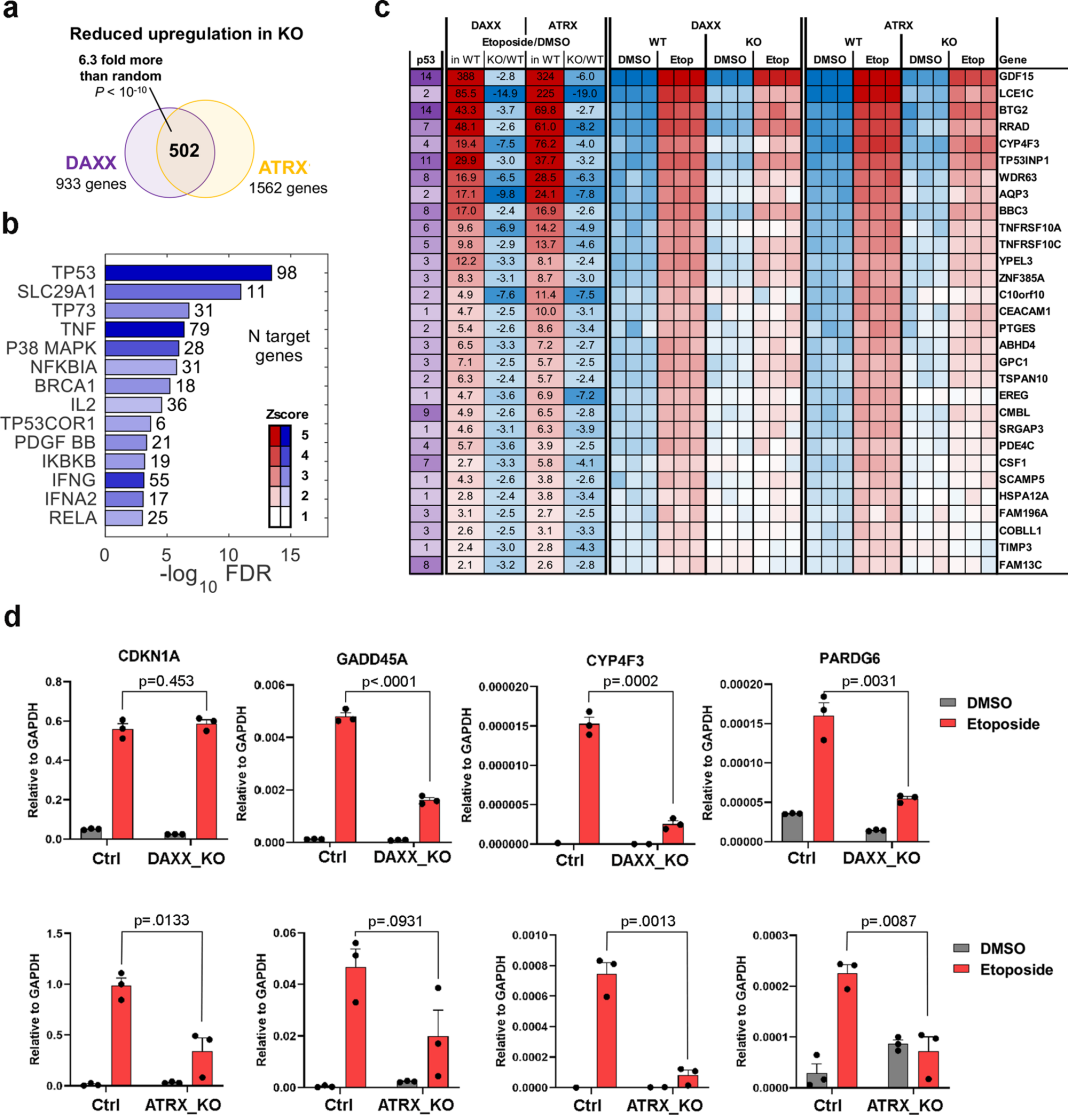
Transcriptomic analysis of DNA damaging response in DAXX_KO and ATRX_KO U87 cells. **a** Venn diagram showing overlap of genes with inhibited effect to treatment with etoposide in DAXX_KO and ATRX_KO relative to WT control cells. **b** Significantly enriched regulators (FDR < 10^−3^, Z > 2) predicted by IPA to have the most inhibited response to etoposide treatment common in both DAXX_KO and ATRX_KO cells. **c** Expression heatmap for top 30 differentially regulated known p53 targets showing a reduction in response to etoposide in DAXX_KO and ATRX_KO relative to WT control. Direct targets of p53 as reported in published genome-wide studies^54^ is indicated in purple. **d** RT-qPCR for DAXX_KO (top row) or ATRX_KO (lower row) relative to Ctrl after DMSO (gray) or 50 μM etoposide (red) treatment. Genes analyzed are indicated, CDKN1A, GADD45A, CYP4F3, and PARDG6. Error bars indicate standard deviation and *P* values determined by two-tailed *t* test for three biological replicates.

We next assayed the RNA-seq (Quantseq) transcriptomic response to DNA damaging agent etoposide in DAXX_KO and ATRX_KO ALT-like cells relative to parental control U87-T cells using RNA-seq (Quantseq) (Fig. 3a). In total, 933 genes in DAXX_KO and 1562 genes ATRX_KO were found to be less responsive to etoposide (upregulated in WT at FDR < 5%, but significantly less upregulated in KO condition at *P* < 0.05). There was an overlap of 502 genes between the two knockout conditions, a 6.4-fold more than expected by chance alone (*P* < 10^−10^, Fisher exact test), indicating very similar functional effect between the two KO cell lines. Enrichment analysis showed several of the most affected regulators (FDR < 10^−3^, Z > 2) including p53 to be significantly inhibited in response to etoposide in DAXX_KO or ATRX_KO cells (Fig. 3b). There was a strong correlation of the magnitude of the attenuation effect between DAXX_KO and ATRX_KO (Spearman *r* = 0.34, *P* < 10^−10^) (Fig. 3c). Among the genes with the most reduced response (at least fourfold, Fig. 3c) were many well-characterized p53-response genes, including GADD45A, TNFRSF10A (TRAIL), MAFB, TP53INP1, USP2, and TIMP3. RT-qPCR demonstrated that some p53-response genes, such as GADD45A, CYP4F3, and PARDG6 were affected by DAXX_KO and ATRX_KO, while other genes, such as CDKN1A, were not significantly affected in DAXX_KO (Fig. 3d). RNA-seq analysis detected no mutations in p53, indicating the reduced p53 response is not due to acquired mutation in the p53 protein-coding sequence.

### Altered p53 chromatin binding in DAXX_KO and ATRX_KO U87 ALT-like cells

**Fig. 4:**
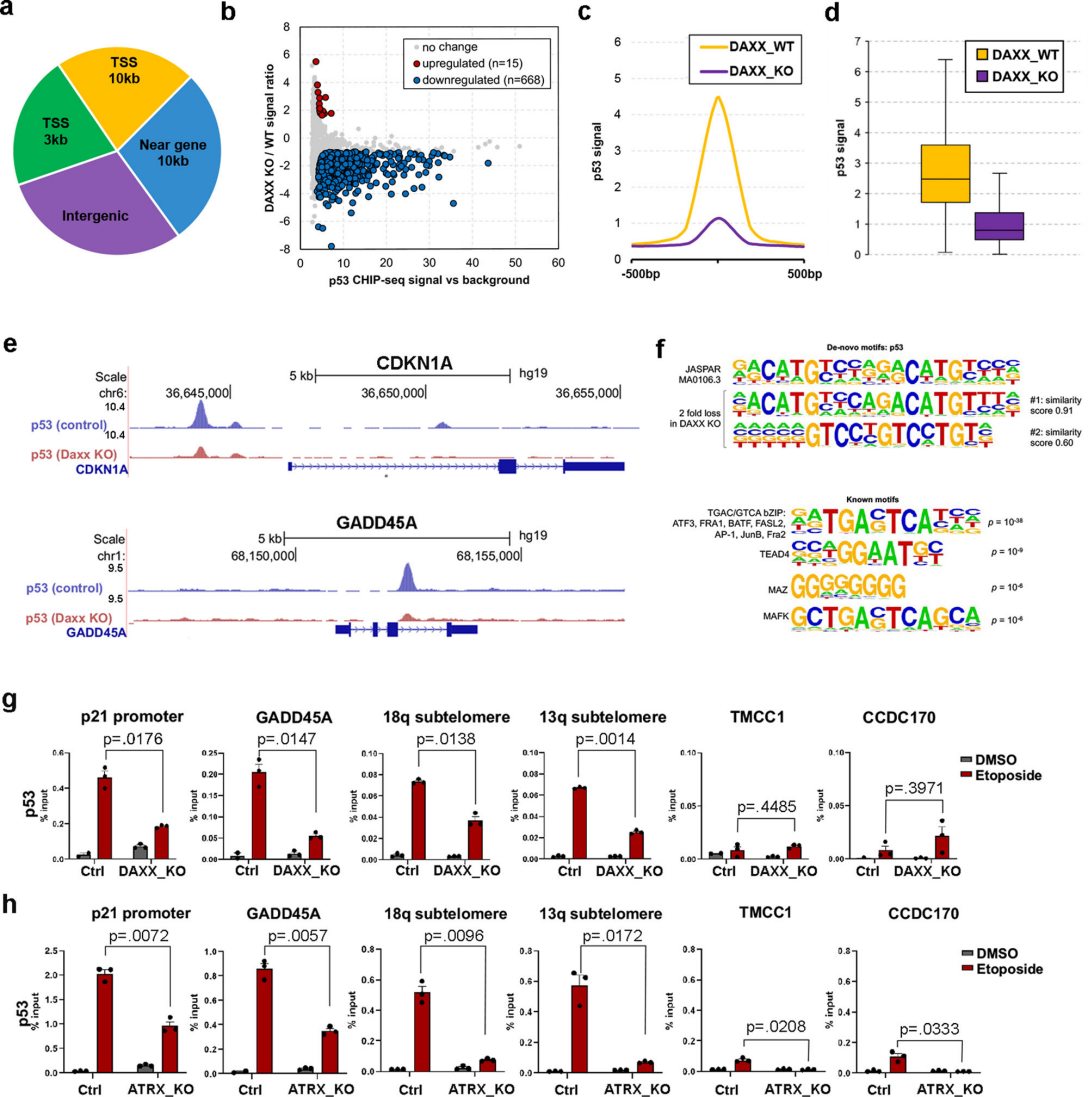
Attenuation of p53 chromatin binding in DAXX_KO and ATRX KO U87 cells. **a** Distribution of p53 peaks within gene context. **b** Downregulated (blue) and upregulated (red) p53 ChIP-seq peaks in DAXX KO relative to WT cells (*y* axis) as a function of p53 ChIP-seq intensity (*x* axis). There is a trend of p53 being downregulated with only few upregulated peaks that are of lower ChIP-seq signal. **c** Mean p53 signal profile comparison in DAXX KO vs WT condition among the set of downregulated peaks. **d** Box plot of the distribution of p53 signal between DAXX KO and WT conditions among the set of downregulated peaks. Whiskers represent maximum and minimum values and box represent upper quartile, median and lower quartile. **e** ChIP-seq tracks for p53 in control (blue) or DAXX_KO (red) at gene loci for CDKN1A (top) and GADD45A (middle). **f** Consensus motifs for p53 ChIP-seq sites with most differential binding between DAXX_KO and WT (top) and for overlapping sites with known factors other than p53. **g** ChIP-qPCR for p53 in DAXX_KO and WT control cells treated with DMSO or 50 μM etoposide (red) for binding sites at the p21 promoter, GADD45A, 18q subtelomere, 13q subtelomere, TMCC1 or CCDC170 gene loci. ChIP-qPCR with IgG control is shown in lower panels. **h** Same as in panel **g**, except for ATRX_KO cells. Error bars are sdm and *P* valued determined by student *t* test for three biological replicates.

Since many p53-response genes were affected in both DAXX_KO and ATRX_KO cells, we assayed p53 binding by ChIP-seq in DAXX_KO and control cells with etoposide treatment (Fig. 4). p53 ChIP-seq peaks were distributed throughout the genome, with the largest distribution occurring >10 K from the transcription start sites (TSS) or at intergenic loci (Fig. 4a). DAXX_KO led to changes in p53 ChIP-seq peaks in both positive and negative directions, with the large majority of strong signal p53 peaks being downregulated in DAXX_KO (Fig. 4b). The mean intensity and overall distribution of strong signal p53 ChIP-seq peaks were reduced in DAXX_KO relative to DAXX_WT (Fig. 4c, d). Integration of ChIP-seq with RNA-Seq identified specific genes co-regulated with changes in neighboring p53-binding sites (Supplementary Fig. 2). Among those downregulated were LIF, LMNA, and WDR43, while upregulated genes included EDF1, METTL7B, and ARHGAP6. We show specific examples of p53-binding sites decreasing at the CDKN1A and GADD45A (Fig. 4e), compared to not affected (MYOB3) or increasing (EDF1) in DAXX_KO cells relative to control (Supplementary Fig. 2c). A consensus motif sequence analysis using JASPAR and HOMER indicated that two subtypes of p53 consensus binding sites were most affected in DAXX_KO cells (Fig. 4f, upper panel). Most p53-binding sites have a consensus sequence, variations of these sequences such as quarter vs half-sites^18^, high-binding cooperativity vs low-binding cooperativity^26^ or non-canonical binding sequences have different outcomes in terms of regulating gene expression. We identified several other consensus binding sites, such as ATF and Forkhead transcription factors Foxf1, FoxL2, and FoxP1 that had weak, but significant enrichments within p53 sites associated with differential gene expression (Fig. 4f, lower panel). We also correlated p53-binding sites with all available ChIP-seq datasets from ENCODE consortium to identify potential overlaps with known transcription factors or histone modifications (Supplementary Fig. 3). Each set of ENCODE peaks was overlapped with p53 peaks and enrichment of overlap within downregulated p53-binding sites was calculated and tested using Fisher exact test. Among the most significantly associated with transcription factors were cFOS, STAT3, cMYC, and RNA PolII, and the most correlated histone modifications were H3K4me1 and H3K4me2 (FDR < 5%, Fisher exact test). We validated by ChIP-qPCR some of these changes in p53 binding in DAXX_KO and control cells in response to etoposide treatment (Fig. 4g). We found that the induction of p53 binding to p21 promoter (CDKN1A), GADD45A, as well as subtelomeric sites at 18q and 13q were abrogated in DAXX_KO cells treated with etoposide relative to its parental U87-T cells. ChIP-qPCR analysis revealed a similar reduction of p53 binding to the target genes after etoposide treatment in ATRX_KO cells relative to U87-T control cells (Fig. 4h, IgG controls shown in Supplementary Fig. 4). Negative control genomic regions like TMCC1 and CCDC170 demonstrate the specificity of the p53 ChIP assay (Fig. 4g, h and Supplementary Fig. 4).

To investigate if this effect on p53 binding is observed in any cancer-derived ALT cells, we assayed U2OS (Supplementary Fig. 5a, b) and GM847 and VA13 (Supplementary Fig. 5c, d), known to have WT p53 protein and an ATRX null background^29,30^. Western blot revealed that p53 protein was stabilized and phosphorylated upon etoposide treatment in U2OS (Supplementary Fig. 5a) and constitutively elevated in GM846 and VA13 (Supplementary Fig. 5c). However, ChIP-qPCR revealed weak (less than twofold) binding of p53 at the response elements in the promoters of CDKN1A and GADD45A and no binding at the subtelomeric response elements in U2OS and GM847 (Supplementary Fig. 5b,d), while VA13 showed some p53 binding to subtelomere repeats (Supplementary Fig. 5d). These data suggest that several cancer-derived ALT cells with ATRX mutation are also compromised for p53 binding to its target sites in response to DNA-damage agents.

### DAXX and ATRX-dependent chromatin accessibility regulates p53-binding patterns

**Fig. 5:**
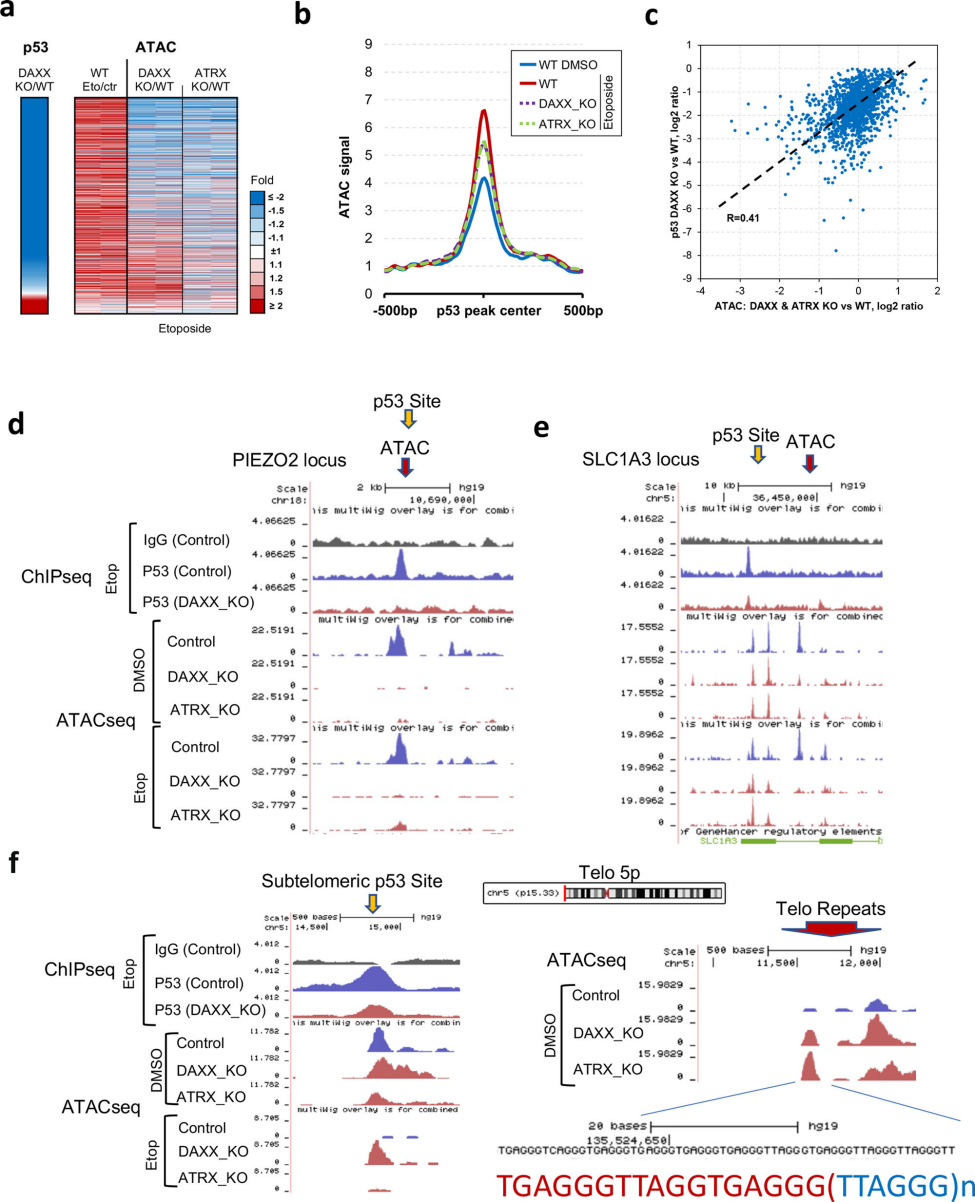
p53 binding correlates with ATAC-Seq changes. **a** Heatmap of ATAC changes at p53 ChIP-seq binding sites, sorted by p53 ChIP-seq signal changes in DAXX KO relative to WT (left). DAXX and ATRX KO are similarly downregulated for open chromatin at p53-binding sites. **b** Line graph showing average ATAC signal around p53 peaks demonstrates upregulation upon etoposide treatment in WT, and that signal gets attenuated in DAXX and ATRX KO. **c** Scatter plot showing a direct correlation between p53-binding reduction in DAXX KO/WT (*y* axis) and ATAC changes in DAXX and ATRX KO/WT under Etoposide treatment (*x* axis). **d**–**f** UCSC browser visualization of p53 ChIP-seq (top) and ATAC-seq data (lower panels) at PIEZO2 (**d**) and SLC1A3 (**e**) and subtelomeric p53 site at telomere 5p (**f**). Telomere repeat sequence is indicated for ATAC-seq peaks at telomere 5p.

To determine if ATRX and DAXX regulate p53 binding through chromatin accessibility, we performed ATAC-seq in WT, DAXX_KO, and ATRX_KO cells treated with etoposide or DMSO control (Fig. 5 and Supplementary Figs. 6 and 7). We found a strong correlation between the reduction in p53 binding with the loss of ATAC-seq peaks (Fig. 5a, b and Supplementary Fig. 6a). ATAC-seq peaks that directly overlap with p53 ChIP-seq peaks followed the same trend of reduced peak intensity in both DAXX_KO and ATRX_KO relative to WT cells (Fig. 5c). We highlight several examples of p53 ChIP-seq and ATAC-seq changes (Fig. 5d–f and Supplementary Fig 6a). At PIEZO2 gene locus, p53-binding site overlaps with ATAC-seq peak in control cells with or without etoposide treatment, and each of these peaks is reduced in DAXX_KO and ATRX_KO (Fig. 5d). We also observed examples of p53-binding loss, with loss of ATAC-seq peak occurring at neighboring sites, with no change to the p53 overlapping peaks, as seen at the SLC1A3 locus (Fig. 5e). We found examples of large chromosome domains (>100 kb) containing p53-binding sites that were uniformly reduced in ATAC-seq peaks, as was observed for a PIEZO2 and ANKFN1 loci (Supplementary Fig 6c, d). We also observed changes at subtelomeric p53-binding sites (Fig. 5f and Supplementary Fig. 7), with the loss of p53 binding corresponding to changes in ATAC-seq, including an increase in peak score for DAXX_KO, but not ATRX_KO. Interestingly, we observed that ATAC-seq peaks in DAXX_KO and particularly ATRX_KO were increased at telomere repeats containing transitions between perfect (TTAGGG)n and imperfect telomere repeat elements (Fig. 5f and Supplementary Fig. 7c). Integration of ATAC-seq with RNA-seq showed a general direct correlation between ATAC-seq peak changes and changes in transcription in DAXX_KO and ATRX_KO cells (Supplementary Fig. 8).

### Rescue of p53 binding by DAXX requires ATRX interaction

**Fig. 6:**
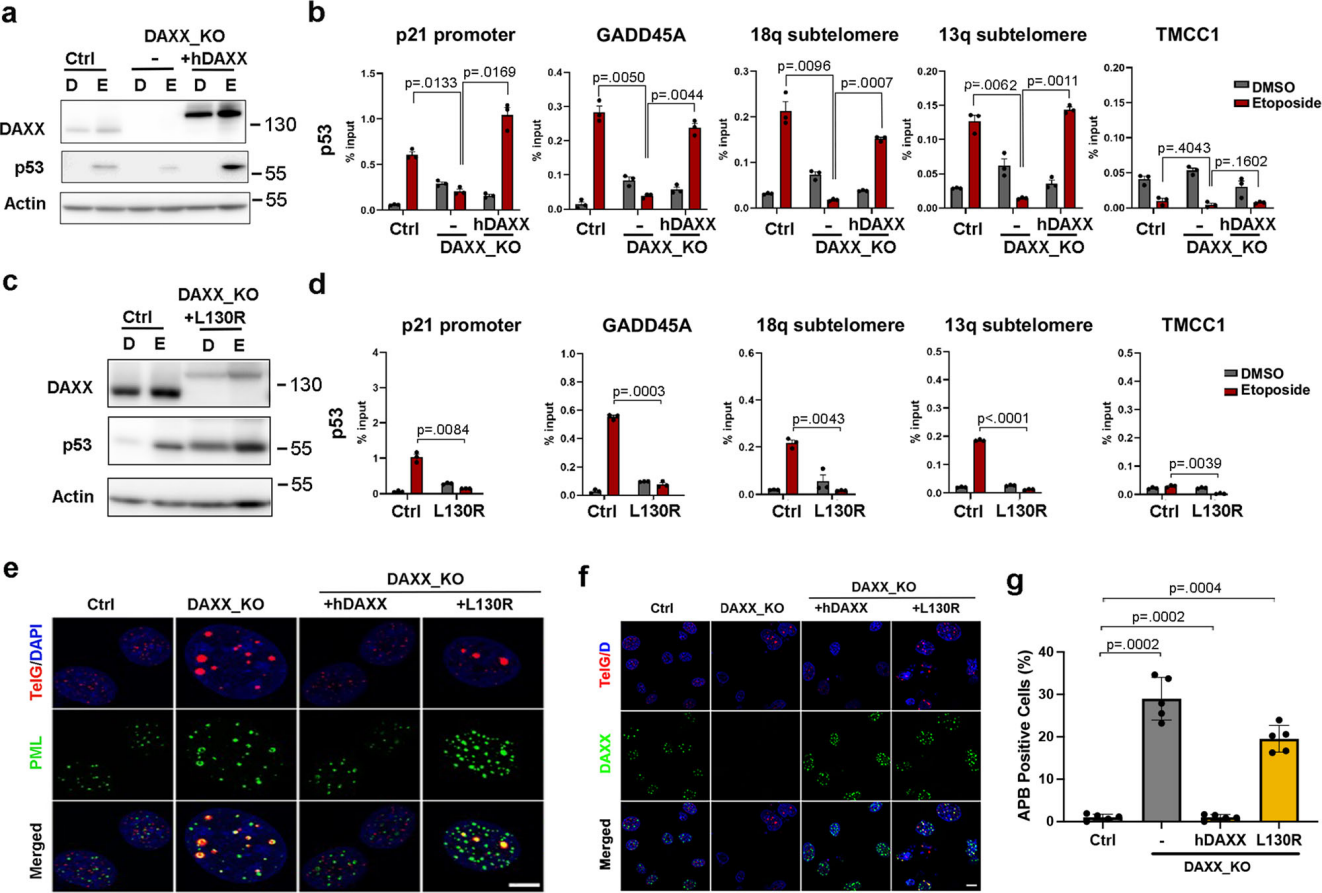
DAXX dependency for p53 function in DAXX_KO U87-T cells. **a** Western blot of Control (Ctrl), DAXX_KO, or DAXX_KO + hDAXX cells treated with DMSO (D) or 50 μM etoposide (E) and probed with antibody to DAXX, p53, or actin. **b** p53 ChIP-qPCR for cells treated as in panel **a**. Error bars indicate standard deviation and *P* values determined by two-tailed student *t* test for three biological replicates. **c** Western blot of Ctrl or DAXX_KO + L130R cells treated with DMSO (D) or etoposide (E) and probed with antibody to DAXX, p53, or actin. **d** p53 ChIP-qPCR for cells treated as in panel **c**. Error bars indicate standard deviation and *P* values determined by two-tailed student *t* test for three biological replicates. **e** IF analysis of Ctrl, DAXX_KO, DAXX_KO + hDAXX, or DAXX_KO + L130R DAXX, imaged for Telomere (TelG, red) + DAPI (blue), PML (green), or merged. Scale bars = 10 μm**. f** IF analysis as described in panel **e**, with DAXX (green), Telomere (TelG, red) + DAPI (blue). Scale bars = 10 μm. **g** Quantification of APBs in Ctrl or DAXX_KO alone, or with hDAXX or L130R DAXX cells. Error bars indicate standard deviation and *P* values determined by two-tailed student *t* test for five biological replicates.

To investigate whether the loss of p53 binding is a specific phenotype of DAXX KO and not an off-target effect of CRISPR gene editing, we utilized a rescue DAXX KO cell line expressing full-length wild-type YFP-tagged hDAXX (hDAXX)^10^ (Fig. 6a). We noticed that DAXX overexpression led to an increase in p53 protein levels in response to etoposide treatment. This suggests p53 protein expression levels may be partly dependent on DAXX. To determine if an interaction between DAXX and p53 may be altered, we assayed this potential interaction by coIP (Supplementary Fig. 9). However, we were unable to demonstrate a stable interaction between p53 and DAXX, although MDM2 was identified in p53 coIP, and ATRX was identified in DAXX coIP (Supplementary Fig. 9). ChIP-qPCR studies showed that the p53 binding at various p53-response elements was defective in DAXX_KO cells, but was restored in the cells expressing hDAXX (Fig. 6b). This was confirmed at the p21 promoter, GADD45A gene, and subtelomeres 18q and 13q. We next tested whether p53 ChIP binding could be rescued by a cancer-associated DAXX mutant previously shown to be deficient for interaction with ATRX (L130R)^10,31^ (Fig. 6c, d). In contrast to wild-type hDAXX, L130R mutant failed to rescue p53 binding at each of the response elements tested (Fig. 6d). We also confirmed that DAXX_KO cells demonstrated ALT-associated PML-nuclear bodies (APBs) with enlarged PML-NBs and colocalization with telomere DNA, as measured by IF and FISH respectively, and this could be suppressed in cells with DAXX WT transgene but not by DAXX L130R mutant transgene (Fig. 6e–g). DAXX_KO cells had elevated telomere C-circles as measured by rolling circle assay, and this was suppressed in cells expressing WT DAXX but not DAXX L130R transgene (Supplementary Fig. 10). These dependencies are consistent with our previous study showing ALT-like features in these and related cell lines with DAXX, as well as ATRX knockouts^10^.

### DAXX and ATRX deficiency alter histone composition at affected p53-binding sites

**Fig. 7:**
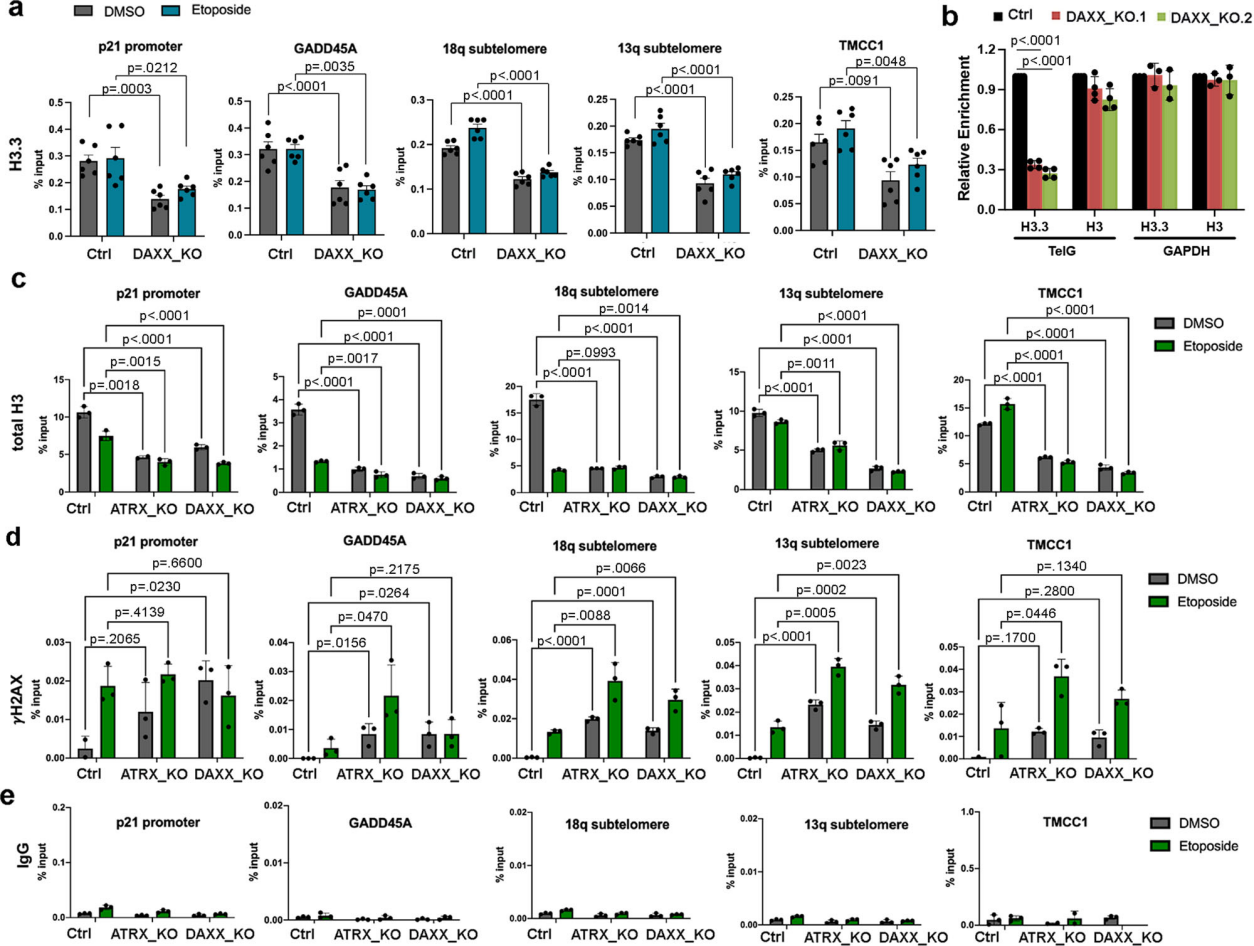
Altered histone composition at p53-binding sites in DAXX _KO and ATRX_KO ALT-like cells. **a** H3.3 ChIP-qPCR in Ctrl or DAXX_KO cells treated with DMSO or etoposide at p21 promoter, GADD45A, 18q subtelomere, 13q subtelomere, or TMCC1 gene loci. **b** ChIP-qPCR assay for H3.3 or H3 probed for telomere DNA or control GAPDH DNA in Ctrl or DAXX_KO cells. **c** Total H3 ChIP-qPCR in Ctrl, ATRX_KO, and DAXX_KO cells treated and assayed as in panel **a**. **d** Histone γH2AX ChIP-qPCR in cells treated and assayed as described in panel **c**. **e** Isotype IgG control ChIP-qPCR for cells treated as described in panels **c** and **d**. Error bars indicate standard deviation and *P* values determined by two-tailed *t* test for three or four (**b**) biological replicates.

To test if DAXX and ATRX KO alter histone composition at p53-binding sites, we first assayed H3.3 binding by ChIP-qPCR. We found that DAXX_KO cells were depleted for histone H3.3 at several p53- binding sites, including those at subtelomeres 13q and 18q (Fig. 7a). We further assayed H3.3 loading at telomere repeats by dot blot analysis, and that DAXX_KO cells had reduced H3.3 at telomere repeats relative to controls, and relative to actively transcribing GAPDH gene (Fig. 7b), consistent with our previous finding that ATRX_KO led to a reduction of H3.3 occupancy at telomeres^10^. We next assessed whether total histone H3 was also affected at these sites (Fig. 7c). We found that total H3 was also reduced in ATRX_KO and DAXX_KO cells at all sites tested, including p53-binding sites at p21, GADD45A, and subtelomere 18q. A similar loss occurred at a non-p53 bound control region TMCC1, suggesting this effect is not restricted to p53-binding sites. Interestingly, etoposide treatment led to a decrease in total H3 in control cells, but not in ATRX_KO or DAXX_KO cells (Fig. 7c). In contrast, we found that these same sites had an increase in γH2AX binding, correspondingly inverse to that observed with total H3 (Fig. 7d). γH2AX was elevated in basal condition in ATRX_KO and DAXX_KO at all sites tested, and had diminished fold increases after etoposide treatment relative to parental control cells (Fig. 7d). No significant background binding was observed with isotype control IgG (Fig. 7e). These findings suggest that DAXX_KO and ATRX_KO ATL-like cells have altered histone composition with diminished histone total H3 and H3.3, and elevated γH2AX at many genomic sites where p53 binding has been compromised.

## Discussion

**Fig. 8:**
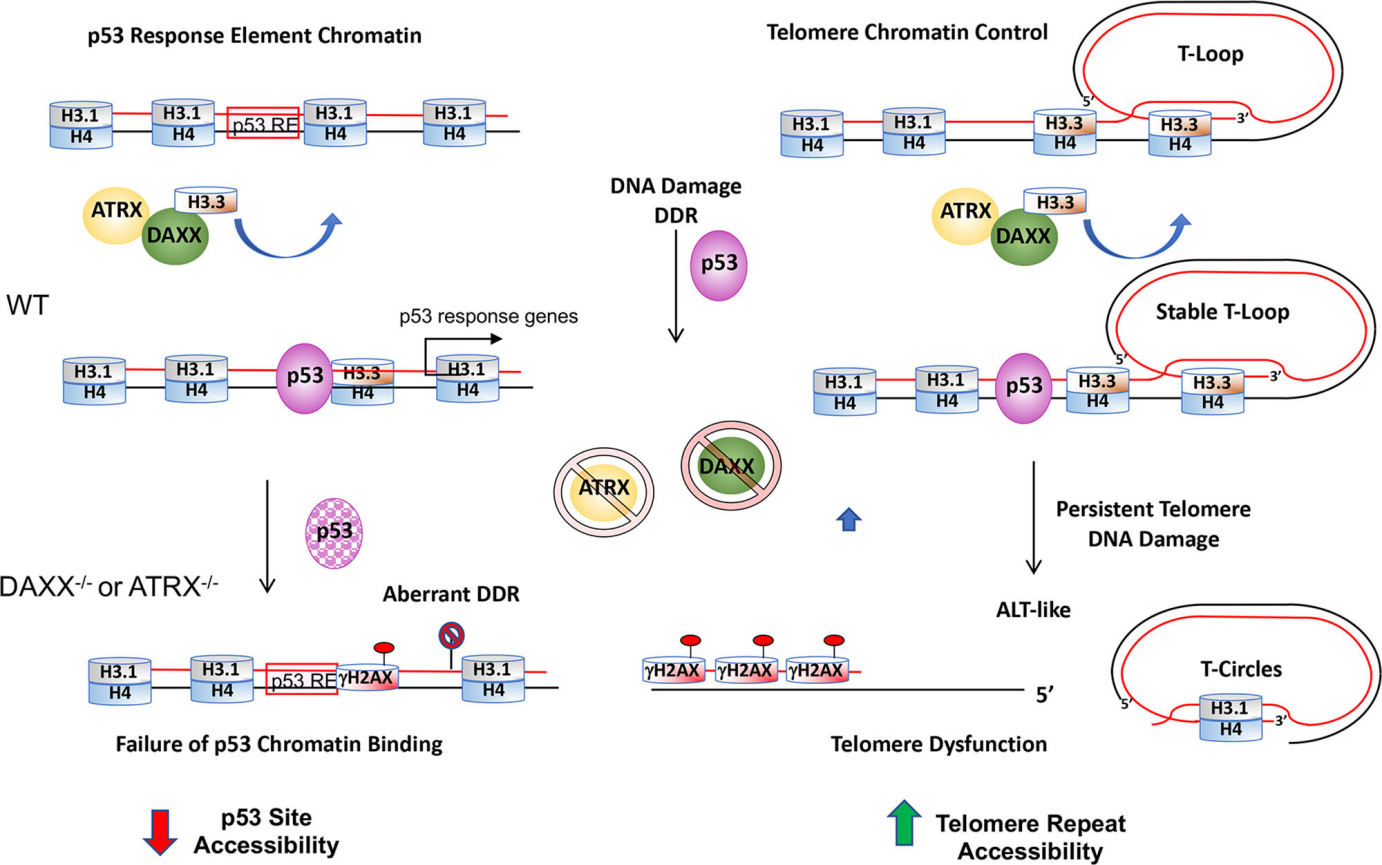
Model of ATRX and DAXX-dependent effects on chromatin accessibility at p53-response elements and telomeres. ATRX and DAXX loss of function mutations lead to a change in p53 chromatin binding and protein stability in U87 glioblastoma cell model. Loss of ATRX or DAXX reduces p53 chromatin binding and transcriptional activation of DNA-damage response genes, such as GADD45A and p21 and aberrant DNA-damage response (DDR). This correlated with a loss of H3.3 binding and accumulation of γH2AX at many loci, including p53-response elements and subtelomeres. Chromatin accessibility was decreased at p53 sites, but increased at telomere repeat DNA, along with loss of telomere T-loops (C-circle formation) and DNA-damage-associated telomere foci (APBs).

DAXX and ATRX function as tumor suppressors that are frequently mutated in ALT cancers, yet their precise role in regulating ALT is not fully elucidated. Here, we show that loss of DAXX or ATRX in a U87-derived glioblastoma cell line gives rise to ALT-like phenotype with corresponding aberrations in the p53-dependent DNA-damage response. We found that ALT-like U87-T cells lacking DAXX or ATRX had increased p53 pathway signaling in the absence of exogenous DNA damaging agents. Paradoxically, these same cells failed to mount a robust p53 response to DNA damage after treatment with etoposide. ATAC-seq studies revealed that loss of ATRX and DAXX alter the landscape of accessible chromatin at many sites that overlapped with p53-response elements, including subtelomeric p53-binding sites and telomere repeat elements themselves. Our findings suggest that loss of DAXX or ATRX result in a loss of chromatin accessibility at p53-binding sites that attenuates the p53 response. Further, loss of DAXX and ATRX alter chromatin structure at many sites, and these changes correlate with reduced total H3 and H3.3 assembly and increased DNA-damage-associated γH2AX formation at numerous genomic loci, including p53-response elements and telomere repeat DNA. We propose that failure of DAXX and ATRX to load H3.3 leads to a global change in chromatin structure, the formation of chronic DNA-damage signaling, attenuated p53 chromatin binding at key sites, and changes in telomeric and subtelomeric chromatin accessibility (Fig. 8). These changes are likely to contribute to the emergence of the ALT cancer pathway.

Both DAXX and ATRX have been implicated in the regulation of p53 and DNA-damage response pathways. DAXX has been shown to co-purify with p53^32^, or associate with p53-regulatory proteins, such as MDM2^33–35^. A recent study has found that DAXX functions as a protein foldase and its interaction with MDM2 regulates p53 functional activity^36^. In a mouse genetic study, heterozygous deletions of DAXX led to an increase sensitivity to low dose radiation and higher incidence of carcinomas, but no overt defect in p53 function or pathway was reported^37^. In a different mouse study, DAXX loss led to the derepression of endogenous retroviral elements (ERVs) that could trigger DNA-damage signaling^38^. In HEK cells, DAXX knockout had little detectable effect on p53 target genes in response to DNA damage^39^. On the other hand, DAXX knockout in PanNET cells led to the derepression of oncogenic drivers, including STC2, which was found be regulated by the DAXX/H3.3/H3K9me3 pathway^40^. The ability to reverse ALT phenotype by the re-introduction of ATRX and DAXX is also context and cell-type dependent^9,10,41^. Furthermore, a recent study found that ATRX chromatin accessibility is cell-type specific and stochastic in single-cell studies^42^. These findings suggest that both DAXX and ATRX regulate the p53 pathway through multiple epigenetic mechanisms.

Our findings indicate that both DAXX and ATRX have related, but non-redundant effects on chromatin accessibility. CRISPR knockout of either DAXX or ATRX U87-T cells had highly concordant effects on chromatin accessibility at p53-binding sites throughout the genome. However, the effects on DAXX and ATRX knockout on chromatin accessibility were complex, with many changes occurring in the absence of exogenous DNA damage, at non-p53-binding sites, and sometimes different for ATRX and DAXX. While chromatin accessibility was typically decreased at strong p53 sites, we found several other sites, including imperfect telomere repeats with increase chromatin accessibility. This is consistent with a proposed role for DAXX and ATRX in telomere heterochromatin and restriction of DNA accessible to ALT-associated DNA damage and recombination. We also observed that DAXX and ATRX KO had common changes in chromatin accessibility associated with large (>100 kb) chromatin domains that often contained p53-binding sites, among other factors and gene transcripts. We do not yet know the mechanism or significance of these large chromatin domain effects, but suggest they may account for some of the tumor-suppressor functions of DAXX and ATRX.

There are several limitations to our study. Most of the work focuses on a single-cell line (U87-T) and CRISPR-deletions generated in this background where ALT phenotypes were selected. Despite this, we did observe many overlapping chromatin and p53 phenotypes in the independent ATRX and DAXX CRISPR-knockout cell lines. We also found similar attenuation of the p53 pathway in several tumor-derived ALT cell lines, including U2OS, GM847, and VA13. Our study can not exclude that non-chromatin-dependent activities may also regulate p53 stability and DNA-binding activity, as we did observe a general decrease in p53 protein abundance in ATRX and DAXX KO cell lines. However, p53 binding was found to be elevated at a small fraction of chromosomal sites (Fig. 4b and Supplementary Fig 2c) suggests that chromosome structure and accessibility, rather than p53 protein stability, are primary regulators of p53 binding. While DAXX-ATRX has been a well-established chaperone for H3.3, and we demonstrate changes in chromatin accessibility and H3.3 loading at many p53 sites, we also observed a similar loss of total H3 and an increase in γH2AX. This suggests that DAXX and ATRX loss leads to a more general defect in histone assembly. The nature of the chromatin structure that blocks p53 binding is not yet known. R-loops and G-quadruplex DNA have been implicated as potential sources of DNA damage in ALT cells^43^. ATRX is important for the suppression of R-loops^44,45^ and G4 quartet formation^46^ and these structures are likely to interfere with p53 binding^47^. In this respect, ATRX and DAXX may regulate additional processes related to chromatin structure and DNA-damage control. Others have shown that mutations in ATRX and DAXX can occur in combination with missense mutations in TP53 in many glioblastomas^48,49^. Thus, it should not be surprising that mutations in p53 accelerate tumorigenesis even in the context of DAXX or ATRX defects that compromised chromatin binding. Furthermore, many TP53 mutations confer gain-of-function activity that affects chromatin accessibility^50^, suggesting that barriers to p53 chromosome binding may be a common mechanism of tumorigenesis.

Our study finds that DAXX and ATRX KO cells with ALT-like phenotype have aberrant p53 DNA-damage response pathways. These cells have an increase in the constitutive activation of p53-response genes, but a failure of p53 to respond to exogenous damage. We note that p73 and p63 are elevated under these conditions and may partly account for the basal activation of the p53 pathway in the absence of p53 functionality due to ATRX or DAXX loss. The loss of p53 functionality correlates with reduced binding to consensus sequences throughout the genome with more drastic effects at some subtelomeric sites. We find that the DAXX-dependent defect in p53 correlates with its interaction with ATRX and the assembly of H3 family of histones on chromatin, including telomeres. We conclude that the DAXX-ATRX complex plays an important role in regulating p53 access to its binding sites and regulation of the p53 DNA-damage response. We propose that this aberrant DNA-damage response is a key contributor to the acquisition of the ALT-like phenotype.

## Methods

### Cell lines

U87 and U2OS cells used in this study were obtained from American Type Culture Collection (ATCC). ALT cell lines GM847 and VA13 were described as p53+ ALT cells previously^17^ and obtained as gift from Roger Greenberg (University of Pennsylvania). U87, U87-T, U87-derived cell lines were described previously^10^. U87-T cells were generated by transducing U87 cells with pBabe-Lox-TERT-Lox. ALT-positive single-cell clones of DAXX_KO and ATRX_KO U87 cells were previously described and characterized^19^. DAXX_KO cells is previously referred to as U87-T 4-17^10^. ATRX_KO is previously referred to as U87-T A-d#1^10^. DAXX_KO cells is generated as previously described^10^. Briefly, U87-T cells were co-transfected with sgRNA pairs targeting hDAXX and a Puro^r^ containing vector, selected with puromycin for 3–5 days, followed by removal of puromycin. Cells were recovered in a regular growth medium, single clones were expanded and screened by immunostaining and Western blot. U87-T sgCon (referred to a control) has empty Cas9 lentivirus vector with TERT transgene. U87-T derived and ALT cells were cultured in DMEM (Life Technologies) supplemented with 10% FBS and penicillin/streptomycin. U2OS cells were cultured in McCoy’s 5 A medium (Life Technologies) supplemented with 10% FBS and penicillin/streptomycin.

### Antibodies and chemicals

The following antibodies were used: p53 DO-1 (sc-126X for ChIP), p53 DO-1 (Millipore OP-43L for western blots), γH2AX JBX-301 (Millipore 05-636), phospho-p53 (Ser-15) (Cell Signaling Technology 9284), histone H3 (ABCAM ab1791), ATRX (Bethyl Laboratories A301-045A), DAXX (Sigma 07-471) and MDM2 (Cell Signaling Technology 86934). Etoposide Sigma (E1383) was dissolved as 50 mM stock solution in DMSO and used at concentrations and times indicated.

### Analysis of cell cycle kinetics

U87-T WT sgControl, DAXX_KO, and ATRX_KO cells were seeded at 1 × 10^5^ cells/well in six-well plates and exposed to etoposide (10 μM) or DMSO control in biological triplicates per each condition. After 24 h, cells were permeabilized with cold, 70% ethanol and resuspended in PBS containing PI (10 mg/mL) and RNAse A solution (100 μg/mL). Flow cytometry was performed on a BD-LSR II (BD Biosciences; Bedford, MA), and FloJo software (Ashland, OR) was used for cell cycle analysis.

### RNA-seq

Total RNA was extracted using Trizol following manufacturer’s instructions. RNA was treated with Turbo DNA-free kit (life technologies) and quality checked with TapeStation (Agilent). Libraries were prepared using Quantseq 3’-mRNA kit (Illumina), and sequencing was performed on Illumina Nextseq500 on the high-output mode in a 75 bp single-end run. RNA-seq data were aligned using STAR^51^ algorithm against hg19 human genome and RSEM v1.2.12 software^52^ was used to estimate read counts using gene information from Ensemble transcriptome version GRCh37.p12. Raw counts were used to estimate significance of differential expression difference between any two experimental groups using DESeq2^53^. Overall gene expression changes were considered significant if passed FDR < 5% threshold. Gene set enrichment analysis was done using QIAGEN’s Ingenuity Pathway Analysis software (IPA, QIAGEN Redwood City,www.qiagen.com/ingenuity) using “Upstream Regulators” options. Most significant regulators (FDR < 5%, unless stated otherwise) that had a significantly predicted activation state (|Z| > 2) were reported. Known p53 target list was derived from a published study^54^.

### ChIP-seq

ChIP-seq was performed as previously described^18^ with certain modifications. Etoposide- (50 μM) treated cells were harvested after 24 h of treatment, fixed with formaldehyde for 10 min followed by quenching with glycine. In total, 25 million cells per sample were sonicated using Covaris sonicator to obtain fragments of size between 100–500 bp. Chromatin was incubated with 10 mg of p53 antibody (Santa Cruz sc-126X) overnight followed by pull-down using protein- G Dynabeads (life technologies). Beads were washed, eluted, and reverse cross-linked overnight. IP DNA was purified using a Sigma PCR purification kit. Quality control was performed on TapeStation (Agilent). Libraries were prepared using Illumina protocol and sequencing 75 bp single end on Illumina Nextseq500 on high-output mode. The data were aligned using bowtie^55^ against hg19 version of the human genome, and HOMER^56^ was used to generate bigwig files and call significant peaks vs input using –style factor option. Peaks that passed FDR < 5% threshold were considered significant. Normalized signals for significant peaks were derived from bigwig files using bigWigAverageOverBed tool from UCSC toolbox^57^ with mean0 option. Fold differences between samples were then calculated and p53 peaks that reduced signal at least twofold were considered as affected by DAXX KO. Genes were associated with peaks based on 3 kb from any transcript TSS threshold.

### ATAC-seq

ATAC-seq was performed essentially as previously described^58,59^. WT, DAXX_KO, and ATRX_KO U87-T cells were treated with DMSO or 50 μM etoposide for 24 h. Then cells were harvested, and ATAC-seq was performed in two biological replicates according to the Omni-ATAC-seq protocol with modifications. Briefly, 1 × 10^5^ cells (>95% viability) were washed in 50 μl cold PBS, spun down at 500 × *g* and 4 °C for 5 min, and resuspended in 50 μl cold ATAC-Resuspension Buffer (RSB) (10 mM Tris-HCl, pH 7.4, 10 mM NaCl, 3 mM MgCl_2_) containing 0.1% IGEPAL CA-630, 0.1% Tween-20 and 0.01% Digitonin. Resuspended cells were kept on ice for 3 min, then washed with 1 ml cold ATAC-RSB containing 0.1% Tween-20 but no IGEPAL CA-630 or Digitonin. Nuclei were pelleted at 500 × *g* and 4 °C for 10 min, resuspended in a 50 μl Tn5 transposase reaction mixture following the manufacturer’s protocol (Illumina Tagment DNA Enzyme and Buffer, Illumina), and incubated at 37 °C for 30 min in a thermomixer with 300 rpm mixing. DNA was purified using a MinElute PCR purification kit (Qiagen) and eluted in 10 μl Elution Buffer for library amplification. Tagmented DNA was PCR amplified using the NEBNext HiFi PCR mastermix (New England Bioloabs) with a universal forward and sample-specific reverse oligo for sample barcoding using the following PCR conditions: initial incubations of 72 °C for 5 min and 98 °C for 30 s, followed by five cycles of 98 °C for 10 s, 63 °C for 30 s, and 72 °C for 1 min. Additional number of cycles was determined for each sample through a “side” qPCR reaction using an aliquot of the PCR as a template to determine the number of cycles needed to reach 1/3 of the max fluorescence. Total cycle numbers ranged from seven to ten cycles. PCR products were run on a 1% agarose gel, regions from ~50 bp to ~1 kb were excised, and DNA was extracted using a gel extraction kit (Qiagen). Purified DNA was submitted to the Wistar Institute Genomics core facility for quality analysis and sequencing. All samples were sequenced on NextSeq500 (Illumina) to generate paired-end 2 × 42 bp reads. ATAC-Seq raw reads were aligned using *bowtie*^55^ against the hg19 version of the human genome, followed by *HOMER*^56^ to generate bigwig files and call significant peaks using the “-style dnase” option. Normalized ATAC signals within p53 sites were derived from bigwig files using *bigWigAverageOverBed* tool from the UCSC toolbox^57^ with mean0 option. Unique ATAC-Seq sites with at least one peak that passed an FDR < 5% threshold in both replicates were considered significant. The significance of pair-wise differences between conditions was estimated using DESeq2^53^. Large chromosomal domains with uniform overall ATAC signal changes were defined as any >100 kb regions with at least 5 significant ATAC sites with at least 70% of those significantly changed by both DAXX and ATRX KO (FDR < 5%, fold > 2).

### ENCODE

All available transcription factor and histone modification data peaks were downloaded from ENCODE and overlapped with the set of p53 peaks. The difference in proportions of overlapped peaks between p53 sites downregulated in DAXX KO vs not downregulated was tested using Fisher exact test and Benjamin–Hochberg procedure was used to correct for multiple testing. ENCODE factors and histones that passed FDR < 5% were clustered using binary distance with average linkage.

### ChIP-qPCR

ChIP-qPCR was performed similar to ChIP-seq with few changes. One million cells per sample were used, and sonication was performed using Diagenode water bath sonicator at a high range 30 s on 30 s off for a total of ~20 min with intermittent incubation on ice to obtain fragment size between 100–500 bp. qPCR primers were designed using NCBI primer blast, and the run was performed on ABI QuantStudio 7. Primers used are listed in Supplementary Data 1.

### qRT-PCR

Total RNA was extracted using Trizol following manufacturer’s instructions. RNA was treated with Turbo DNA-free kit (Life Technologies). Primers were designed using NCBI primer blast and the run was performed on ABI QuantStudio 7. Primers used are listed in Supplemental Data 1.

### C-circle assay

C-circle assay was performed as described previously^10^. Briefly, sample DNA digested with *Alu*I and *Mbo*I (30 ng/10 μl) was combined with 10 μl reaction mix [0.2 mg/ml BSA, 0.1% Tween, 0.2 mM each dATP, dGTP, dTTP, 2× ɸ29 Buffer, and 7.5 U ɸDNA polymerase (NEB)]. The reactions were incubated at 30 °C for 8 h, and then at 65 °C for 20 min. The reaction products were diluted to 400 μl with 2× SSC, dot-blotted onto a GeneScreen Plus membrane, and hybridized with a ^32^P-labeled (CCCTAA)_4_ probe at 37 °C for overnight to detect C-circle amplification products. The blots were washed four times at 37 °C in 0.5× SSC/0.1% SDS buffer, exposed to Phosphor-imager screens, visualized by Typhoon 9410 Imager (GE Healthcare Life Sciences), and quantified with ImageQuant 5.2 software (Molecular Dynamics).

### Immuno-FISH assay

Indirect immunofluorescence (IF) combined with fluorescence in situ hybridization (FISH) analysis was performed as described previously^10^. Primary antibody to PML (ab96051, Abcam) was prepared in 1:500 dilution in blocking solution. After IF, cells were fixed in 4% paraformaldehyde in 1× PBS for 10 min, washed in 1× PBS, dehydrated in ethanol series (70%, 95%, 100%), and air-dried. Coverslips were denatured for 5 min at 80–85 °C in a hybridization mix [70% formamide, 10 mM Tris-HCl, pH 7.2, and 0.5% blocking solution (Roche)] containing telomeric PNA-Tamra-(CCCTAA)_3_ probe, and hybridization was continued for 2 h at room temperature in the dark moisturized chambers. Coverslips were washed, counterstained with 0.1 μg/ml DAPI in blocking solution, and mounted with VectorShield (Vector Laboratories). Images were captured with a ×63 lens on a Zeiss LSM780 confocal laser scanning microscope (Carl Zeiss). Cells with >5 PML foci colocalizing with telomere DNA foci were scored as APB positive. The quantification was generated from at least three independent Immuno-FISH experiments.

### Reporting summary

Further information on research design is available in the Nature Research Reporting Summary linked to this article.

## Supplementary information

Supplementary Information

Description of Additional Supplementary Files

Supplementary Data 1

Reporting Summary

## Data Availability

The data supporting the findings of this study are included within the article and its Supplementary Information files. All Illumina-based sequencing data are available from the NCBI Sequence Read Archive (SRA) under accession number code PRJNA773790 with the specific accession code PRJNA773796 for RNA-seq, PRJNA773795 for ChIP-seq, and PRJNA853364 for ATAC-seq datasets. Source data are provided with this paper.

## Source data

Source Data
