# Supplementary material for: DAXX-ATRX regulation of p53 chromatin binding and DNA damage response
Source: Nat Commun. 2022 Aug 26;13:5033. doi: 10.1038/s41467-022-32680-8 (PMC9418176; doi:10.1038/s41467-022-32680-8)
Supplement: Supplementary file 1 — Supplementary Information [file 41467_2022_32680_MOESM1_ESM.pdf]

SUPPLEMENTARY INFORMATION

DAXX-ATRX Regulation of p53 Chromatin Binding and DNA Damage Response

Nitish Gulve[1], Chenhe Su[1,], Zhong Deng[1], Samantha S. Soldan[1], Olga Vladimirova[1], Jayamanna Wickramasinghe[1], Hongwu Zheng[2], Andrew V. Kossenkov[1], and Paul M Lieberman[1*]

Supplementary Figure 1.

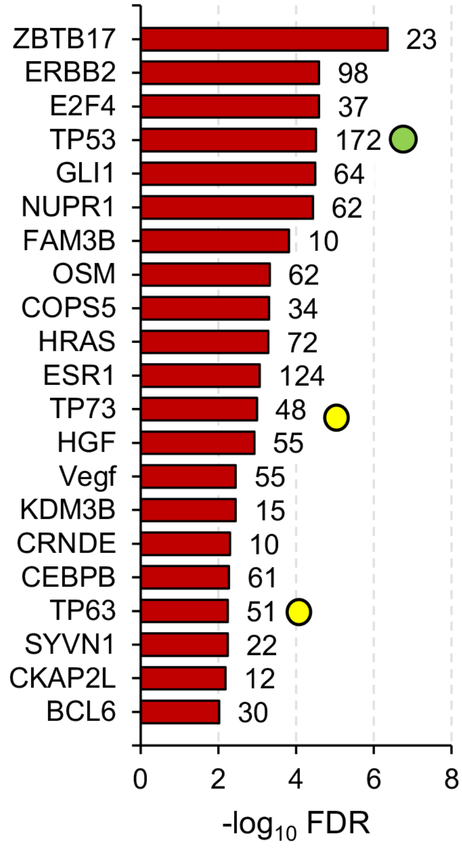

Supplementary Figure 1. P53 Pathway Perturbed in ATRX mutated GBMs. Transcription expression data was analyzed from TCGA GBM dataset consisting of 152 samples that had both whole exome sequencing and RNA-seq data. Of the 152 patients there were 10 with truncating or missense mutation in ATRX**.** 1356 genes were differentially expressed in ATRX mutant vs WT TCGA samples**.** Enrichment analysis of those genes using Ingenuity revealed 21 regulators with significant enrichment (at least 10 targets, FDR<1%) of their known target genes among the list **T**P53 was top 4 by significance and top 1 by number of targets among that list. TP73 and TP63 were also among the highly significant regulators. We also found that all 10 ATRX mutant GBM samples had mutations in TP53.

Supplementary Figure 2

a

DAXX KO/WT p53 fold	DAXX KO/WT mRNA fold	DAXX KO/WT mRNA FDR	Gene
5.9	1.6	0%	EDF1
3.8	1.6	0%	COG6
3.8	1.5	1%	METTL7B
3.6	1.9	1%	IGF1R
3.5	36.7	1%	ARHGAP6
3.4	4.2	0%	ARHGEF25
3.4	1.9	1%	ITGB3
3.4	4.5	0%	NPR2
2.8	1.7	0%	PCNXL2
2.7	1.7	0%	FAM167A
2.6	4.1	0%	EXD3
2.2	2.5	0%	SLC25A35
2.2	1.7	0%	DEAF1

(mRNA heatmap columns: WT 1 2 3, KO 1 2 3) IgG

b

DAXX KO/WT p53 fold	DAXX KO/WT mRNA fold	DAXX KO/WT mRNA FDR	Gene
-10.0	-2.1	0%	C17orf53
-9.8	-1.6	0%	SRRD
-9.8	-1.7	0%	TFIP11
-7.6	-3.5	0%	LIF
-7.5	-1.8	0%	SNRPN
-6.0	-2.4	0%	WDR43
-5.7	-1.4	0%	BLOC1S2
-5.4	-1.4	0%	LMNA
-5.0	-1.3	4%	CCDC57
-4.6	-1.6	4%	DYRK3
-4.5	-1.5	0%	BTBD10
-4.4	-1.8	0%	VWCE
-4.2	-2.6	0%	RGS20

(mRNA heatmap columns: WT 1 2 3, KO 1 2 3)

c

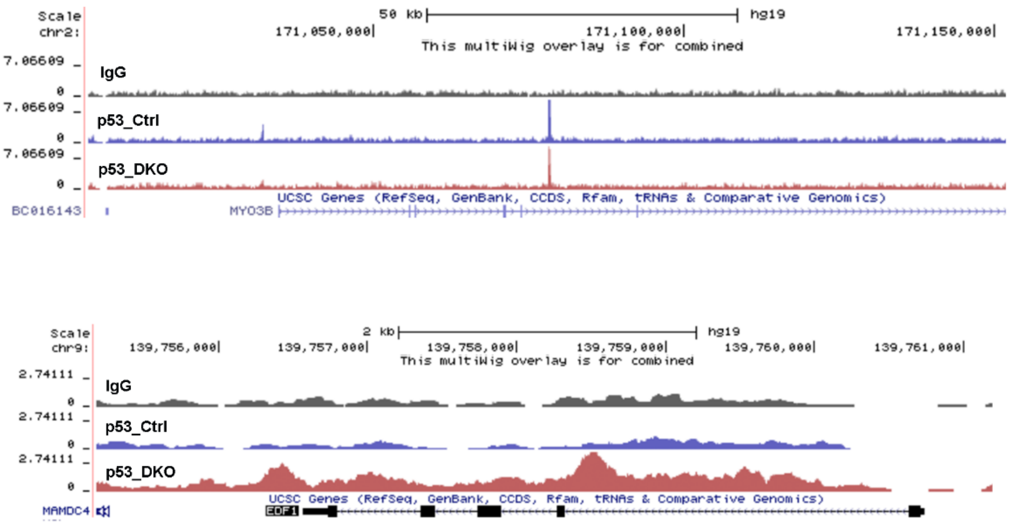

Supplementary Figure 2. Integrative analysis of ChIP-seq with RNA-seq for p53 binding and transcription regulation. a-b. Top coding genes with p53 binding changes resulting in mRNA changes for genes up-regulated **(a)** or down-regulated **(b)** by DAXX_KO.

c. UCSC genome browser visualization of ChIP-seq tracks for IgG and p53 from Ctrl or DAXX_KO cells treated with etoposide showing minority examples of p53 sites not affected (Myo3B) or up-regulated (EDF1) by DAXX_KO.

Supplementary Figure 3.

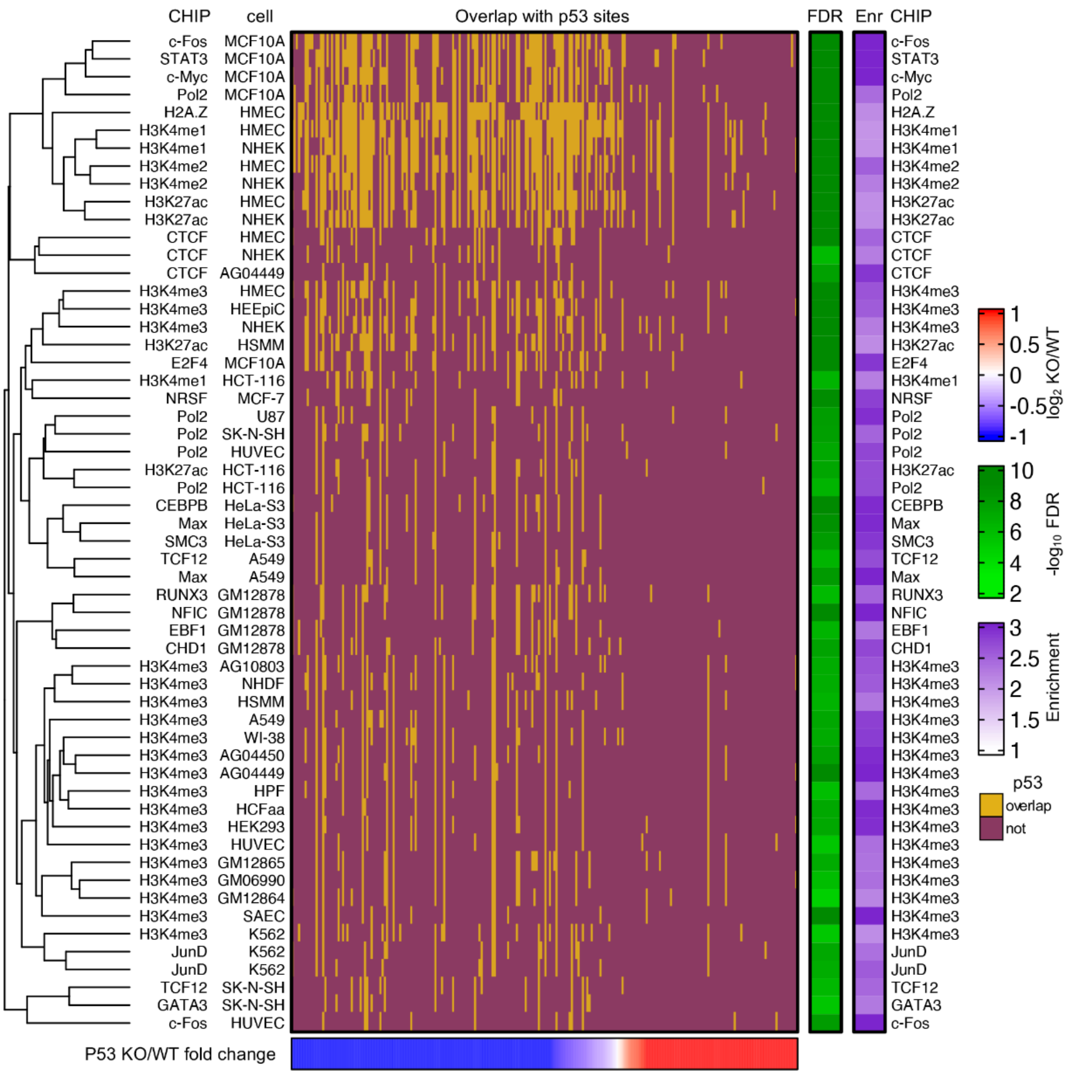

Supplementary Figure 3. ENCODE data transcription factors and histones shown to be enriched within p53 binding sites that reduce signal after DAXX KO. Main orange heatmap indicates presence of corresponding factor or histone mark from ENCODE (each row) at the site occupied by p53 (column) in our model that was downregulated upon DAXX KO. p53 sites are arranged by magnitude of change in DAXX KO/WT (red-white-blue scale). Enrichment (purple) of overlap within downregulated p53 peaks and their significance FDR (green) values are indicated.

Supplementary Figure 4.

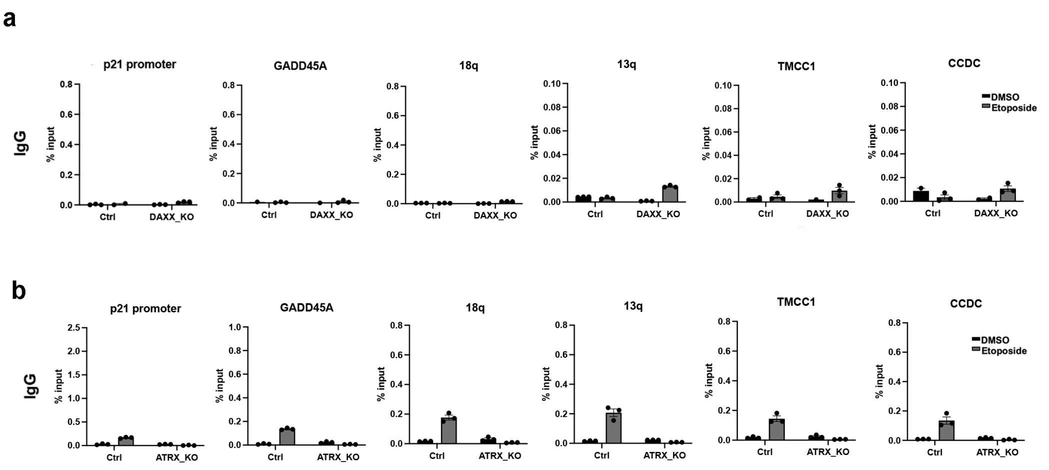

Supplementary Figure 4. Isotype IgG control ChIP-qPCR for experiments shown in Figure 4g and h.

Supplementary Figure 5.

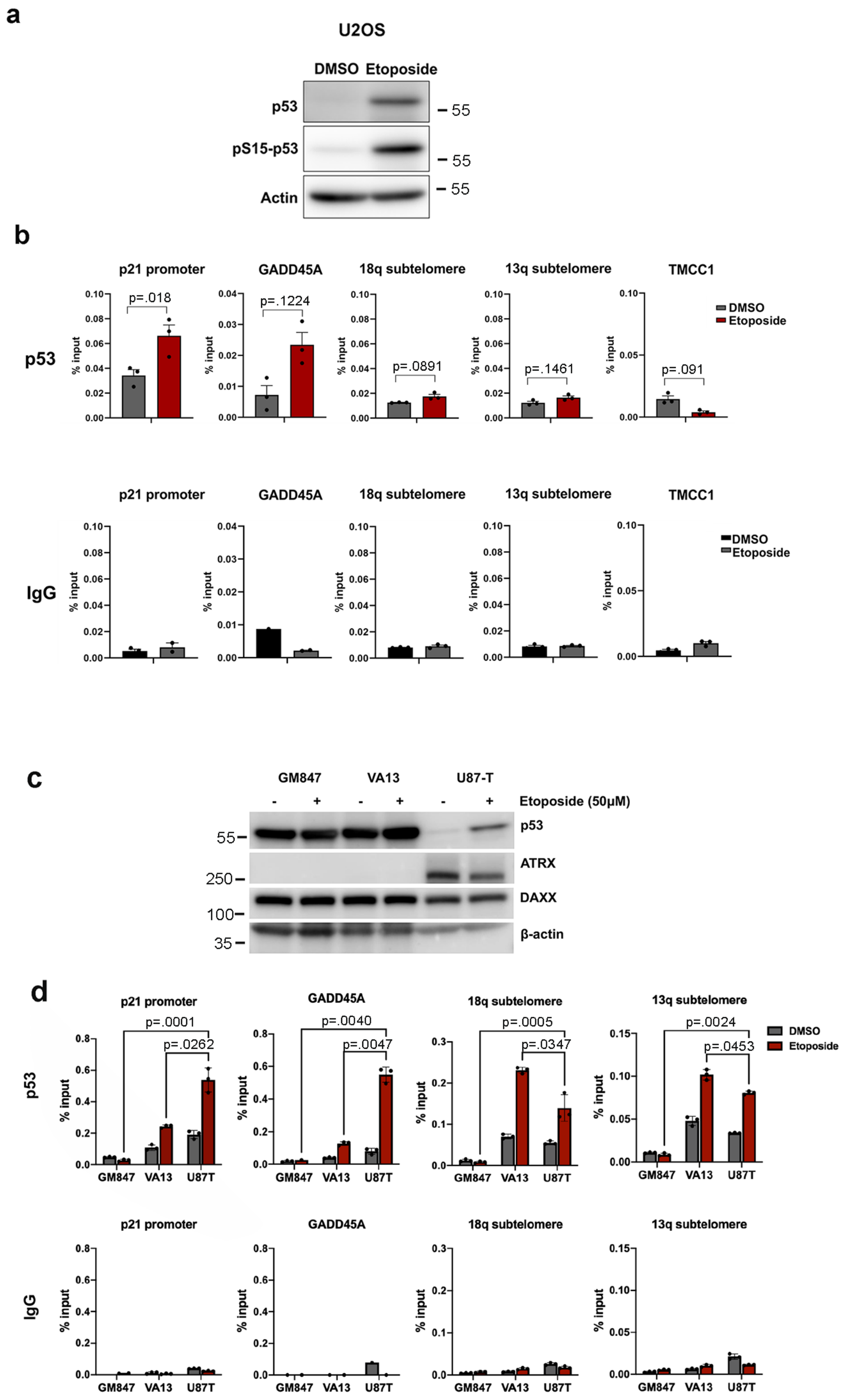

Supplementary Figure 5. **P53 analysis in ALT cells. a.** Western blot of U2OS cells treated with DMSO or 50 μM etoposide for 24 hr, assayed with antibody to p53, pS15-p53, or Actin. **b**. ChIP assay for U2OS cells treated as in panel **a** with either DMSO (grey) or etoposide (red) using antibody to p53 (top row) or IgG control (lower row), and assayed by qPCR with primers for p53 binding sites at the p21 promoter, GADD45A, 18q subtelomere, 13q subtelomere, or TMCC1. **c.** Western blot of GM847, VA13, or U87-T cells treated with DMSO or 50 μM etoposide for 24 hr, assayed with antibody to p53, ATRX, DAXX, or Actin. **d**. ChIP assay for GM487, VA13, or U87-T cells treated as in panel **c** with either DMSO (grey) or etoposide (red) using antibody to p53 (top row) or IgG control (lower row), and assayed by qPCR with primers for p53 binding sites at the p21 promoter, GADD45A, 18q subtelomere, or 13q subtelomere. Error bars indicate standard deviation and p-values determined by two-tailed t-test for 3 biological replicates.

Supplementary Figure 6.

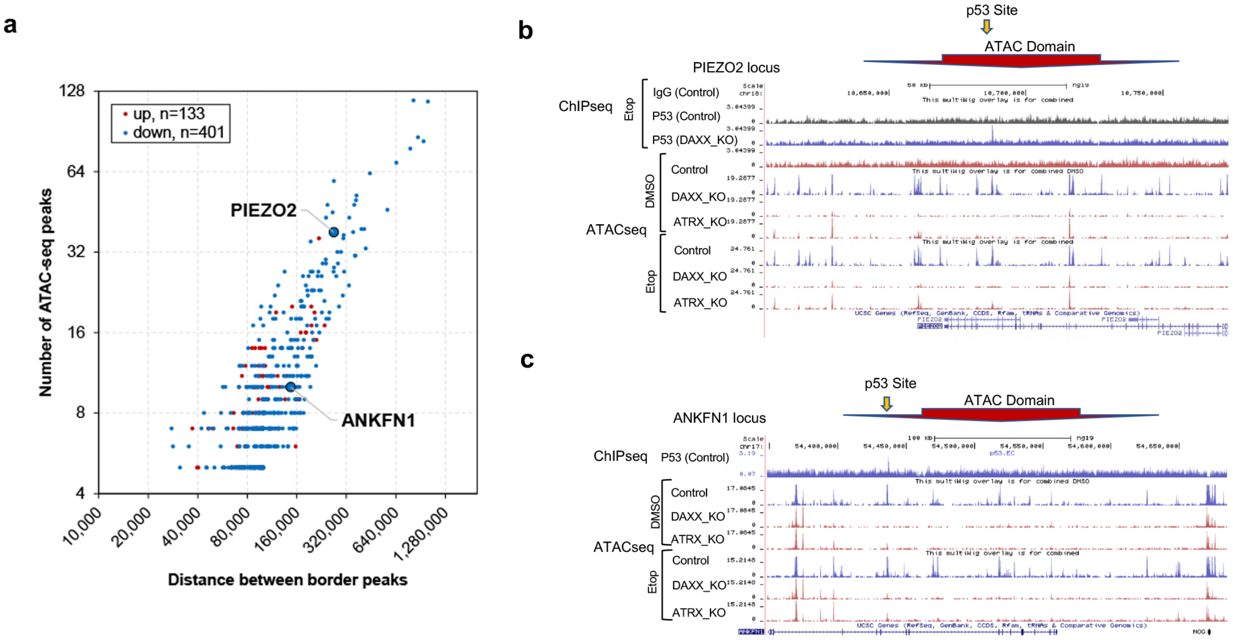

Supplementary Figure 6. Correlations of p53 ChIP-seq and ATAC-seg large chromomsome domains. **a.** Domains of >100kb with at least 5 ATAC-seq peaks, 70% of which are significantly changed in both DAXX and ATRX_KO conditions (FDR<5%, at least 2 fold). Distance between border ATAC peaks (can be <100kb) versus total number of ATAC-seq peak in the domain. **b-c**. UCSC browser visualization of >100 kb domains at PIEZO2 (**b**) and AKNF1a (**c**) gene loci. P53 peaks are indicated by arrows, and large ATAC-domains are highlighted by red bar above each panel.

Supplementary Figure 7.

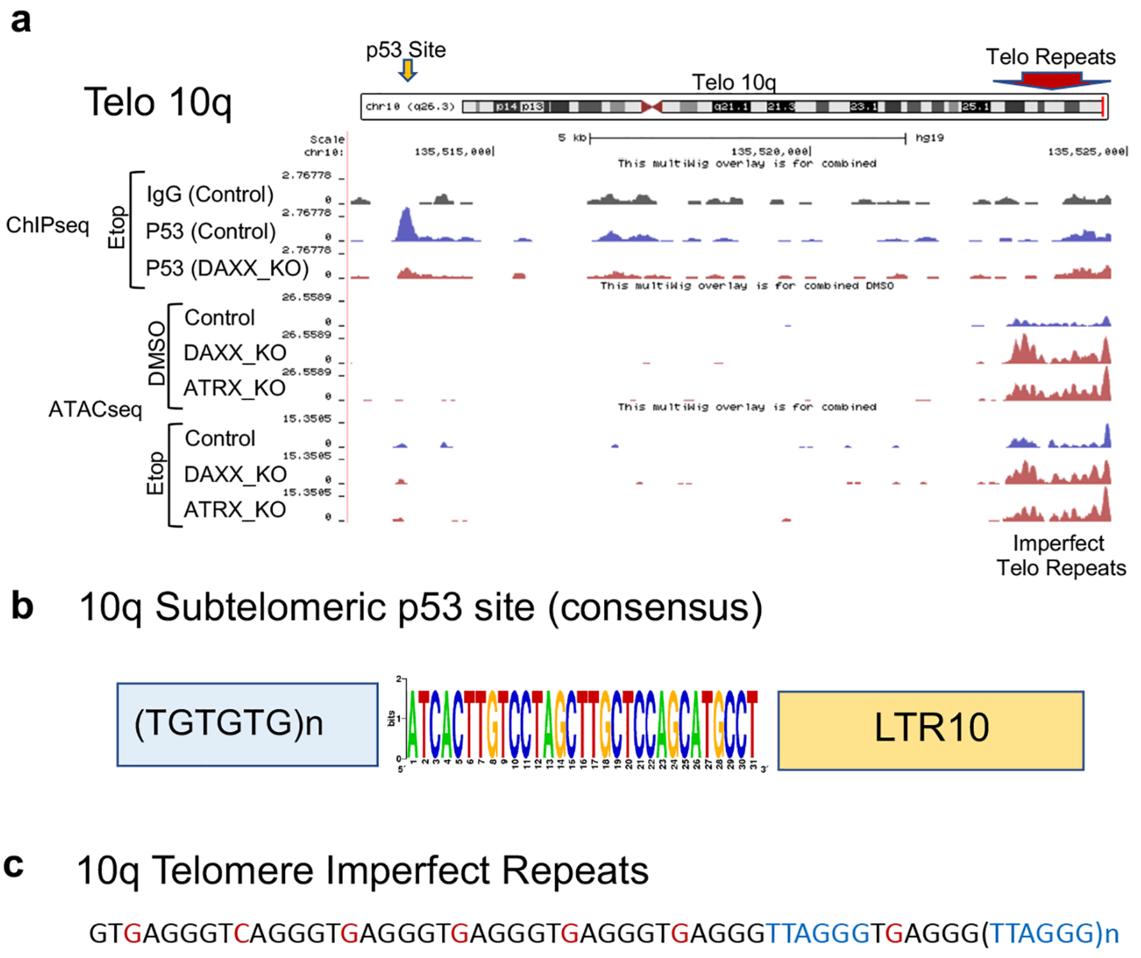

Supplementary Figure 7. DAXX_KO and ATRX_KO alter subtelomeric p53 binding and ATAC-seq peaks at telomere 10q. **a**. UCSC browser visualization of ChIP-seq (top) and ATAC-seq (bottom) data. ChIP-seq for etoposide treated for IgG control and p53 control or DAXX_KO. ATAC-seq for DMSO or etoposide treated control, DAXX_KO or ATRX_KO as indicated. Arrow for p53 binding site and telomere repeats are indicated above. **b.** Analysis of the subtelomeric p53 binding site in 10q showing (TGTGTG)n repeat at 5' and LTR10 element 3' with consensus p53 site highlighted with Jaspar generated color scheme. **c**. Imperfect telomere repeat elements transition to perfect (TTAGGG)n repeats show increase ATAC-seq peak in ATRX and DAXX_KO cells.

Supplementary Figure 8.

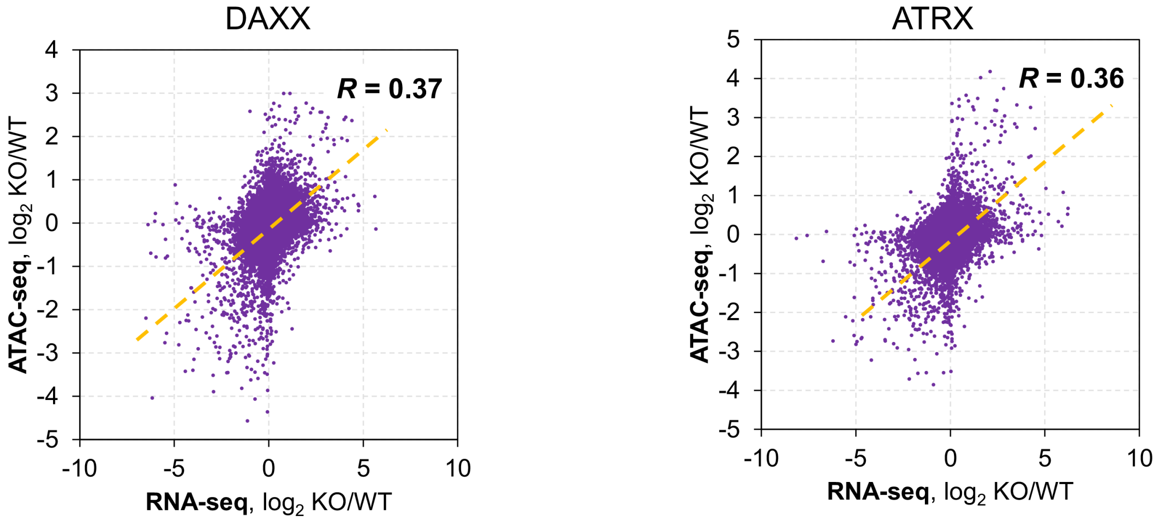

Supplementary Figure 8. Correlation of ATAC-seq with RNA-seq.

ATAC signal was measured in DMSO condition for 500bp window round the 5'-most transcript's TSS. Expressed genes with detected ATAC signal are used (n=22,547) RNAseq changes are correlated with ATAC signal changes in DMSO conditions.

Supplementary Figure 9.

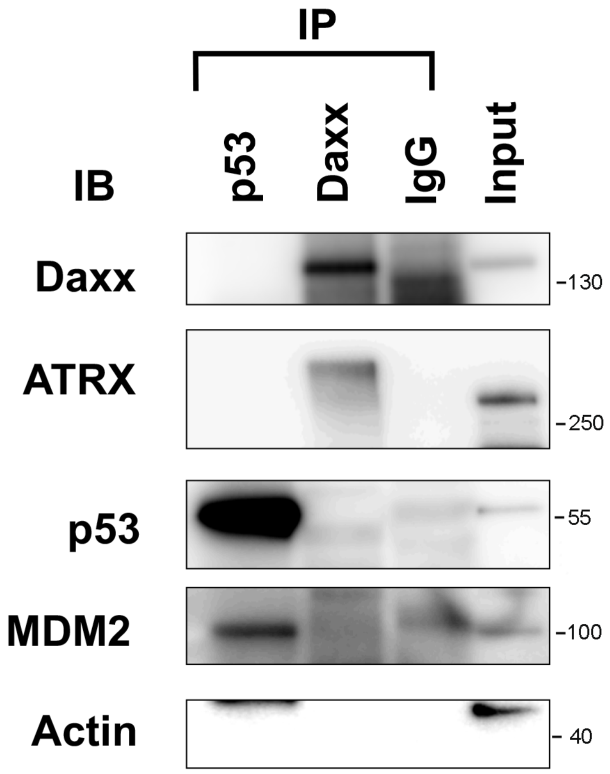

Supplementary Figure 9. **CoIP of p53 with MDM2 but not DAXX or ATRX in U87 cells.** U87T control cells were subject to IP with p53, DAXX, or IgG control, and then assayed by Western blot for DAXX, ATRX, p53 or MDM2. Input (5%) is show for each antibody.

Supplementary Figure 10.

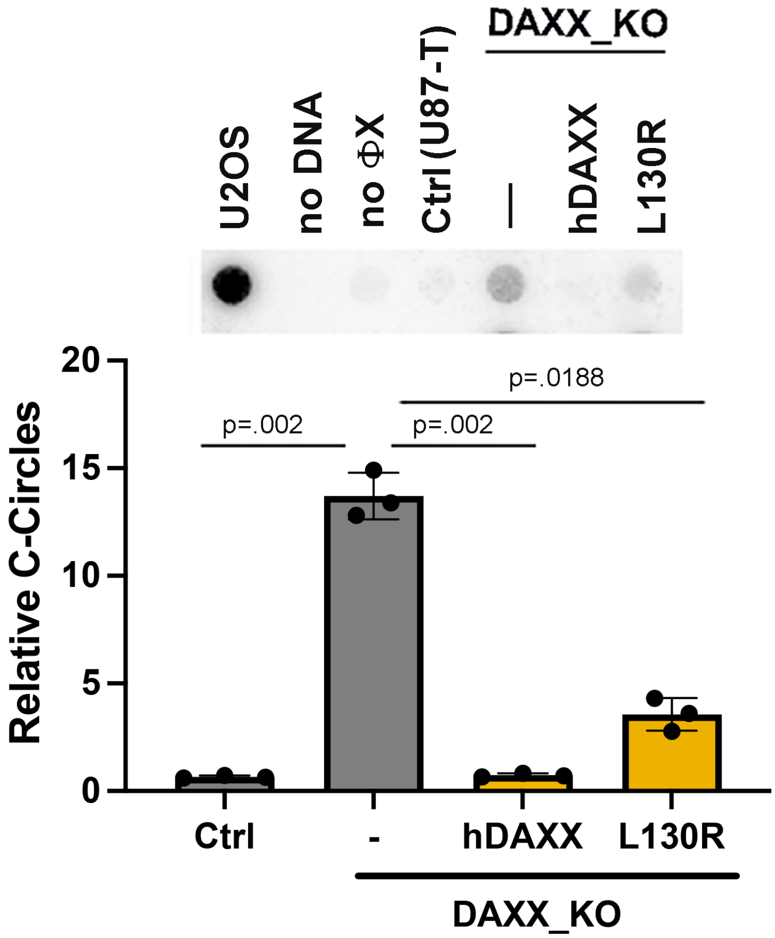

Supplementary Figure 10. C-circle assay showing representative data (insert) and quantification of Ctrl or DAXX_KO alone, or with hDAXX or L130R DAXX cells. p value determined by two-tailed student t-test for 3 biological replicates.
