# Supplementary material for: DAXX-ATRX regulation of p53 chromatin binding and DNA damage response
Source: Nat Commun. 2022 Aug 26;13:5033. doi: 10.1038/s41467-022-32680-8 (PMC9418176; doi:10.1038/s41467-022-32680-8)
Supplement: Supplementary file 2 — Description of Additional Supplementary Files [file 41467_2022_32680_MOESM2_ESM.pdf]

Description of Additional Supplementary Files

File Name: Supplementary Data 1
Description: List of primers used in this study.
