# Supplementary material for: DAXX-ATRX regulation of p53 chromatin binding and DNA damage response
Source: Nat Commun. 2022 Aug 26;13:5033. doi: 10.1038/s41467-022-32680-8 (PMC9418176; doi:10.1038/s41467-022-32680-8)
Supplement: Supplementary file 4 — Reporting Summary [file 41467_2022_32680_MOESM4_ESM.pdf]

nature portfolio

Corresponding author(s): Paul M. Lieberman

Last updated by author(s): 02/04/2022

Reporting Summary

Nature Portfolio wishes to improve the reproducibility of the work that we publish. This form provides structure for consistency and transparency in reporting. For further information on Nature Portfolio policies, see our Editorial Policies and the Editorial Policy Checklist.

Statistics

For all statistical analyses, confirm that the following items are present in the figure legend, table legend, main text, or Methods section.

n/a	Confirmed	
☐	☒	The exact sample size (n) for each experimental group/condition, given as a discrete number and unit of measurement
☐	☒	A statement on whether measurements were taken from distinct samples or whether the same sample was measured repeatedly
☐	☒	The statistical test(s) used AND whether they are one- or two-sided<br>*Only common tests should be described solely by name; describe more complex techniques in the Methods section.*
☒	☐	A description of all covariates tested
☒	☐	A description of any assumptions or corrections, such as tests of normality and adjustment for multiple comparisons
☐	☒	A full description of the statistical parameters including central tendency (e.g. means) or other basic estimates (e.g. regression coefficient) AND variation (e.g. standard deviation) or associated estimates of uncertainty (e.g. confidence intervals)
☐	☒	For null hypothesis testing, the test statistic (e.g. F, t, r) with confidence intervals, effect sizes, degrees of freedom and P value noted<br>*Give P values as exact values whenever suitable.*
☒	☐	For Bayesian analysis, information on the choice of priors and Markov chain Monte Carlo settings
☒	☐	For hierarchical and complex designs, identification of the appropriate level for tests and full reporting of outcomes
☒	☐	Estimates of effect sizes (e.g. Cohen's d, Pearson's r), indicating how they were calculated

Our web collection on statistics for biologists contains articles on many of the points above.

Software and code

Policy information about availability of computer code

Data collection	no software was used
Data analysis	STAR, v2.7.0<br>RSEM, v1.2.12<br>bigWigAverageOverBed, v2<br>DESeq2, v1.30.1<br>HOMER, v4.8.3<br>Ingenuity Pathway Analysis, v73620684

For manuscripts utilizing custom algorithms or software that are central to the research but not yet described in published literature, software must be made available to editors and reviewers. We strongly encourage code deposition in a community repository (e.g. GitHub). See the Nature Portfolio guidelines for submitting code & software for further information.

Data

Policy information about availability of data

All manuscripts must include a data availability statement. This statement should provide the following information, where applicable:

- Accession codes, unique identifiers, or web links for publicly available datasets
- A description of any restrictions on data availability
- For clinical datasets or third party data, please ensure that the statement adheres to our policy

The RNA-seq, CHIP-seq and ATAC-seq data were submitted to the Gene Expression omnibus (GEO) database and can be accessed using accession number: GSE186443

Field-specific reporting

Please select the one below that is the best fit for your research. If you are not sure, read the appropriate sections before making your selection.

☒ Life sciences ☐ Behavioural & social sciences ☐ Ecological, evolutionary & environmental sciences

For a reference copy of the document with all sections, see nature.com/documents/nr-reporting-summary-flat.pdf

Life sciences study design

All studies must disclose on these points even when the disclosure is negative.

Sample size	Cells were counted using automated cell counter. Identical number of cells were used in each sample in all experiments.
Data exclusions	No data was excluded.
Replication	All experiments were performed in biological duplicates or triplicates. All replications were successful.
Randomization	There are primarily two treatment conditions that were used - DMSO and Etoposide treatment. And three types of cells- control, DAXX knockout and ATRX knockout were tested in the two treatments.
Blinding	Blinding was not relevant for the study.

Reporting for specific materials, systems and methods

We require information from authors about some types of materials, experimental systems and methods used in many studies. Here, indicate whether each material, system or method listed is relevant to your study. If you are not sure if a list item applies to your research, read the appropriate section before selecting a response.

Materials & experimental systems

n/a	Involved in the study	
☐	☒	Antibodies
☐	☒	Eukaryotic cell lines
☒	☐	Palaeontology and archaeology
☒	☐	Animals and other organisms
☒	☐	Human research participants
☒	☐	Clinical data
☒	☐	Dual use research of concern

Methods

n/a	Involved in the study	
☐	☒	ChIP-seq
☐	☒	Flow cytometry
☒	☐	MRI-based neuroimaging

Antibodies

Antibodies used	p53 ChIP/IP sc-126X santacruz<br>p53 western blot OP43-100ug Millipore<br>PML IP sc-966X santacruz<br>PML western blot A301-167A Bethyl<br>ATRX western blot A301-045A Bethyl<br>gH2AX ChIP/western blot 05-636 Millipore<br>H3.3 ChIP/western blot 09-838 Millipore
Validation	Validation statements are available on the manufacturer's websites.

Eukaryotic cell lines

Policy information about cell lines

Cell line source(s)	U87T Cell lines were engineered in a previous study. [https://doi.org/10.15252/embj.201796659]<br>U2OS cells were obtained from ATCC.
Authentication	Cell lines were authenticated by STR analysis.
Mycoplasma contamination	Cell lines were routinely tested for Mycoplasma contamination. All cell lines were Mycoplasma negative.
Commonly misidentified lines<br>(See ICLAC register)	*Name any commonly misidentified cell lines used in the study and provide a rationale for their use.*

ChIP-seq

Data deposition

☒ Confirm that both raw and final processed data have been deposited in a public database such as GEO.

☒ Confirm that you have deposited or provided access to graph files (e.g. BED files) for the called peaks.

Data access links<br>*May remain private before publication.*	https://www.ncbi.nlm.nih.gov/geo/query/acc.cgi?acc=GSE186443<br>reviewer token cbapusekrdenjgt
Files in database submission	p53.WT.fastq fastq for CHIP-seq of p53, DAXX WT cells<br>p53.KO.fastq fastq for CHIP-seq of p53, DAXX KO cells<br>IgG.WT.fastq fastq for CHIP-seq of IgG, DAXX WT cells<br>IgG.KO.fastq fastq for CHIP-seq of IgG, DAXX KO cells<br>input.WT.fastq fastq for CHIP-seq of input, DAXX WT cells<br>input.KO.fastq fastq for CHIP-seq of input, DAXX KO cells<br>p53.WT.bw: bigwig graph tracks<br>p53.KO.bw: bigwig graph tracks<br>IgG.WT.bw: bigwig graph tracks<br>IgG.KO.bw: bigwig graph tracks<br>input.WT.bw: bigwig graph tracks<br>input.KO.bw: bigwig graph tracks
Genome browser session<br>(e.g. UCSC)	http://genome.ucsc.edu/cgi-bin/hgTracks?hgsid=1195426927_kuv3mH6DgChT6glbQFRvheaWvSxs

Methodology

Replicates	p53 CHIP-seq. samples were run using one replicate.
Sequencing depth	75bp single end read run produced following number of reads<br>sample reads<br>p53.WT 100,280,519<br>p53.KO 75,334,300<br>IgG.WT 68,628,572<br>IgG.KO 70,335,502<br>input.WT 58,423,260<br>input.KO 63,139,129
Antibodies	p53 ChIP/IP sc-126X santacruz
Peak calling parameters	default parameters with -style factor option, FDR<5%, fold>4 criteria
Data quality	FDR<5%, fold>4 criteria were used to call peaks versus corresponding igg or input. Numbers of peaks:<br>p53.WT vs igg 1195<br>p53.WT vs input 1154<br>p53.KO vs igg 697<br>p53.KO vs input 768
Software	HOMER v4.8.3

Flow Cytometry

Plots

Confirm that:

☒ The axis labels state the marker and fluorochrome used (e.g. CD4-FITC).

☒ The axis scales are clearly visible. Include numbers along axes only for bottom left plot of group (a 'group' is an analysis of identical markers).

☐ All plots are contour plots with outliers or pseudocolor plots.

☒ A numerical value for number of cells or percentage (with statistics) is provided.

Methodology

Sample preparation	U87-T WT sgControl, Daxx_KO, and ATRX_KO cells were seeded at 1 x 105 cells/well in 6-well plates and exposed to etoposide (10 M) or DMSO control in biological triplicates per each condition. After 24h, cells were permeabilized with cold, 70% ethanol and resuspended in PBS containing PI (10 mg/mL) and RNAse A solution (100 g/mL). Flow cytometry was performed on a BD-LSR II (BD Biosciences; Bedford, MA) and FloJo software (Ashland, OR) was used for cell cycle analysis.
Instrument	*Identify the instrument used for data collection, specifying make and model number.*
Software	*Describe the software used to collect and analyze the flow cytometry data. For custom code that has been deposited into a community repository, provide accession details.*
Cell population abundance	*Describe the abundance of the relevant cell populations within post-sort fractions, providing details on the purity of the samples and how it was determined.*
Gating strategy	*Describe the gating strategy used for all relevant experiments, specifying the preliminary FSC/SSC gates of the starting cell population, indicating where boundaries between "positive" and "negative" staining cell populations are defined.*

☐ Tick this box to confirm that a figure exemplifying the gating strategy is provided in the Supplementary Information.
