# Supplementary material for: DAXX-ATRX regulation of p53 chromatin binding and DNA damage response
Source: Nat Commun. 2022 Aug 26;13:5033. doi: 10.1038/s41467-022-32680-8 (PMC9418176; doi:10.1038/s41467-022-32680-8)
Supplement: Supplementary file 5 — Source Data [file 41467_2022_32680_MOESM5_ESM.zip › Source_data.pdf]

Figure 2a

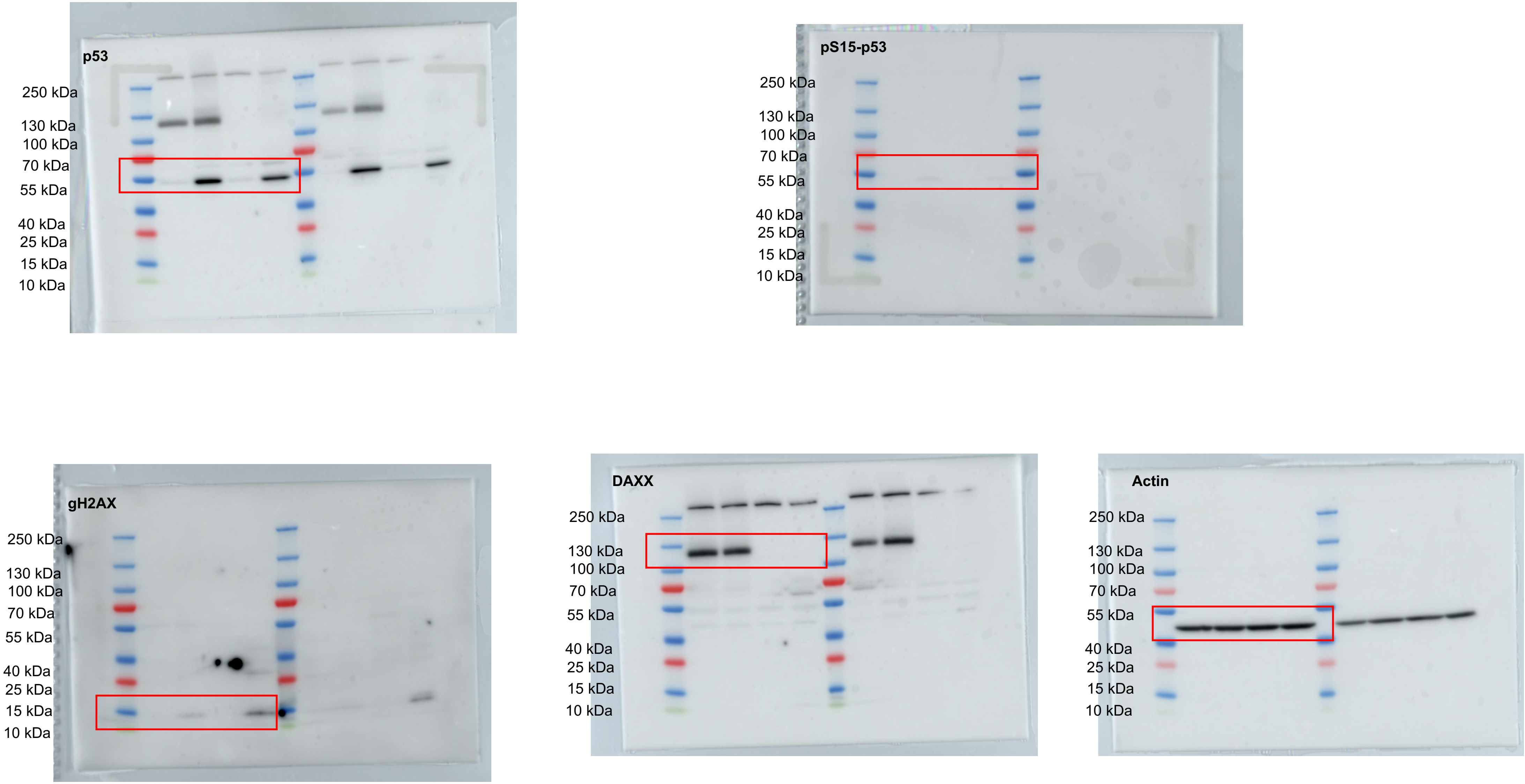
p53
250 kDa
130 kDa
100 kDa
70 kDa
55 kDa
40 kDa
25 kDa
15 kDa
10 kDa
pS15-p53
250 kDa
130 kDa
100 kDa
70 kDa
55 kDa
40 kDa
25 kDa
15 kDa
10 kDa
gH2AX
250 kDa
130 kDa
100 kDa
70 kDa
55 kDa
40 kDa
25 kDa
15 kDa
10 kDa
DAXX
250 kDa
130 kDa
100 kDa
70 kDa
55 kDa
40 kDa
25 kDa
15 kDa
10 kDa
Actin
250 kDa
130 kDa
100 kDa
70 kDa
55 kDa
40 kDa
25 kDa
15 kDa
10 kDa

Figure 2b

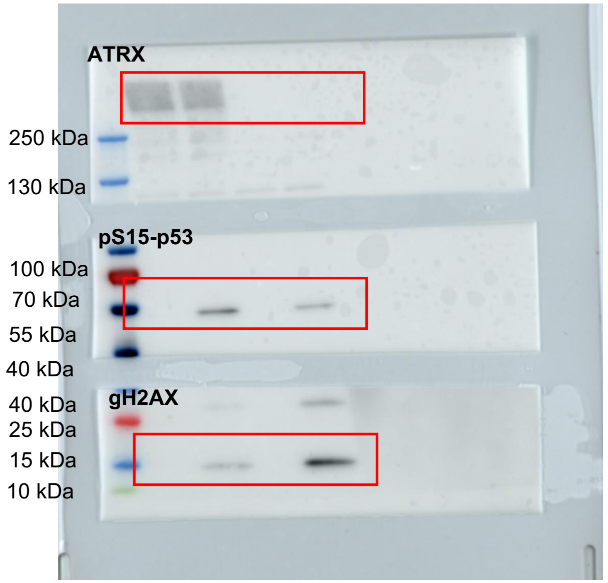
ATRX
250 kDa
130 kDa
pS15-p53
100 kDa
70 kDa
55 kDa
40 kDa
40 kDa
gH2AX
25 kDa
15 kDa
10 kDa

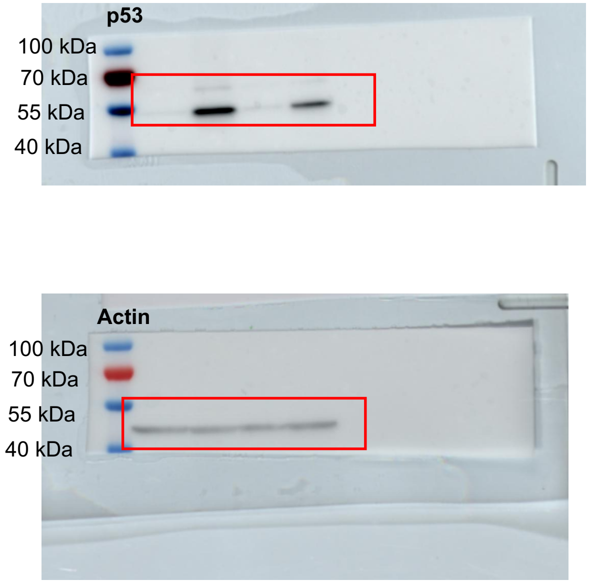
p53
100 kDa
70 kDa
55 kDa
40 kDa
Actin
100 kDa
70 kDa
55 kDa
40 kDa

Figure 6a

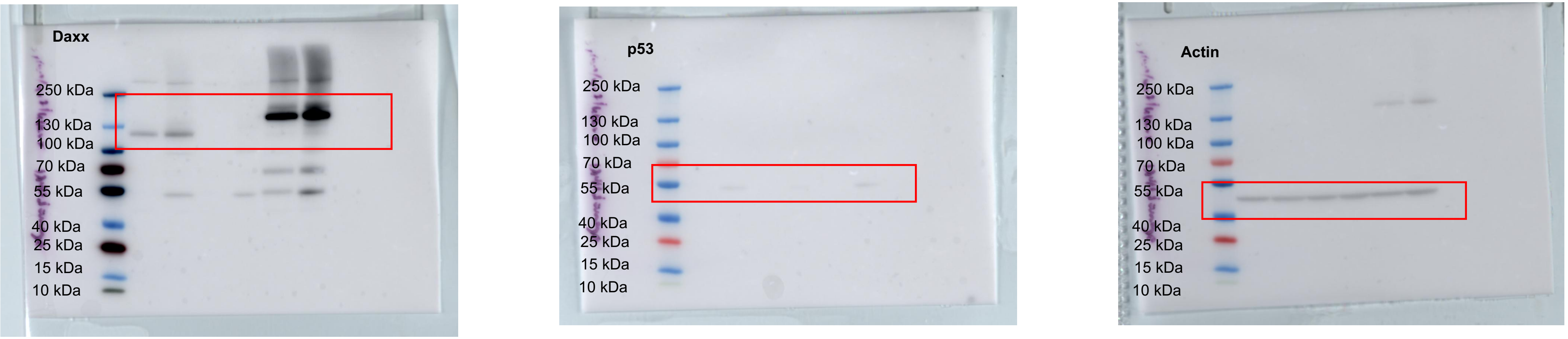
Daxx
250 kDa
130 kDa
100 kDa
70 kDa
55 kDa
40 kDa
25 kDa
15 kDa
10 kDa
p53
250 kDa
130 kDa
100 kDa
70 kDa
55 kDa
40 kDa
25 kDa
15 kDa
10 kDa
Actin
250 kDa
130 kDa
100 kDa
70 kDa
55 kDa
40 kDa
25 kDa
15 kDa
10 kDa

Figure 6c

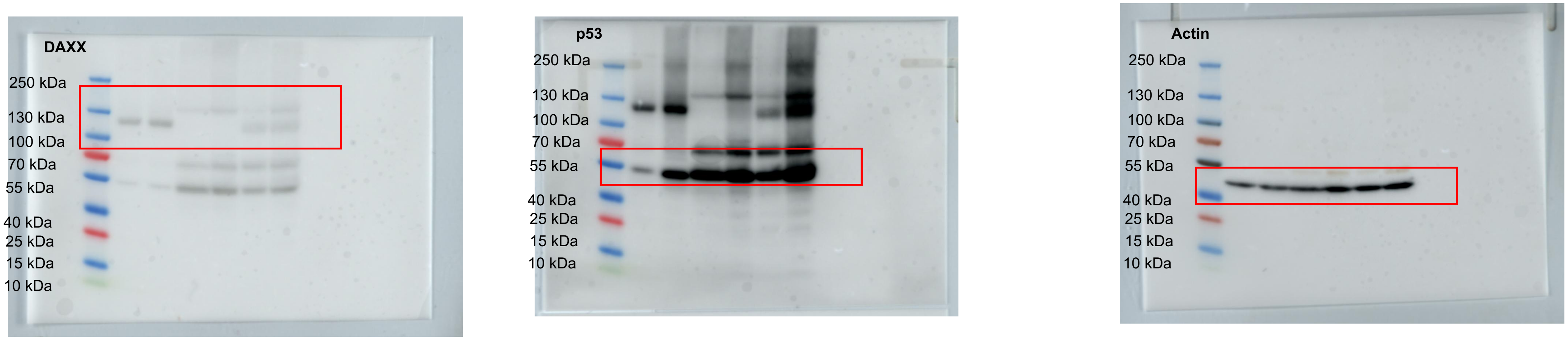
DAXX
250 kDa
130 kDa
100 kDa
70 kDa
55 kDa
40 kDa
25 kDa
15 kDa
10 kDa
p53
250 kDa
130 kDa
100 kDa
70 kDa
55 kDa
40 kDa
25 kDa
15 kDa
10 kDa
Actin
250 kDa
130 kDa
100 kDa
70 kDa
55 kDa
40 kDa
25 kDa
15 kDa
10 kDa

Supplementary Figure 5a

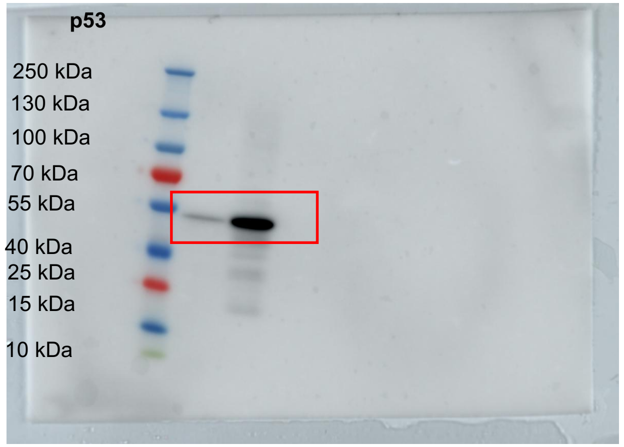

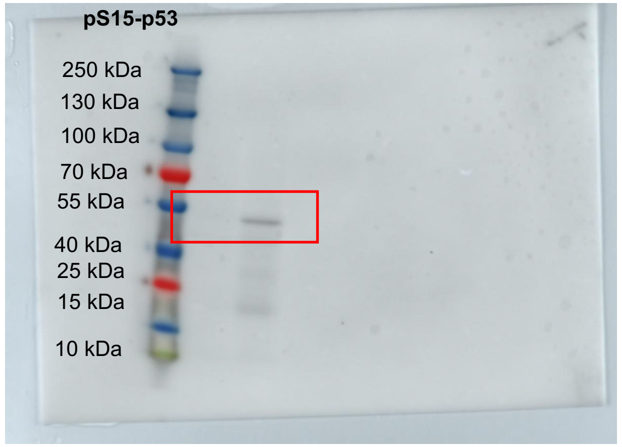

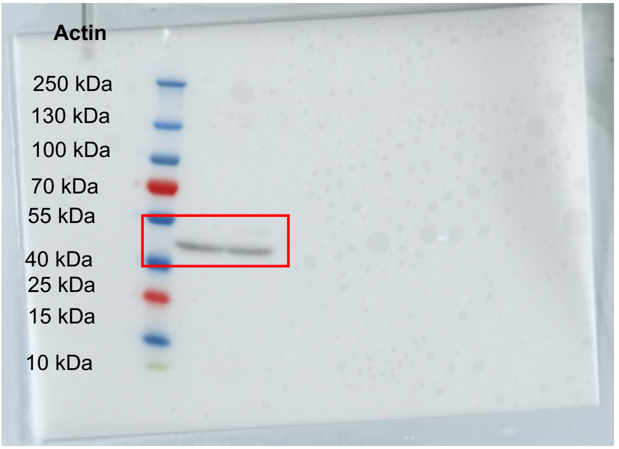

Supplemental Fig.5c

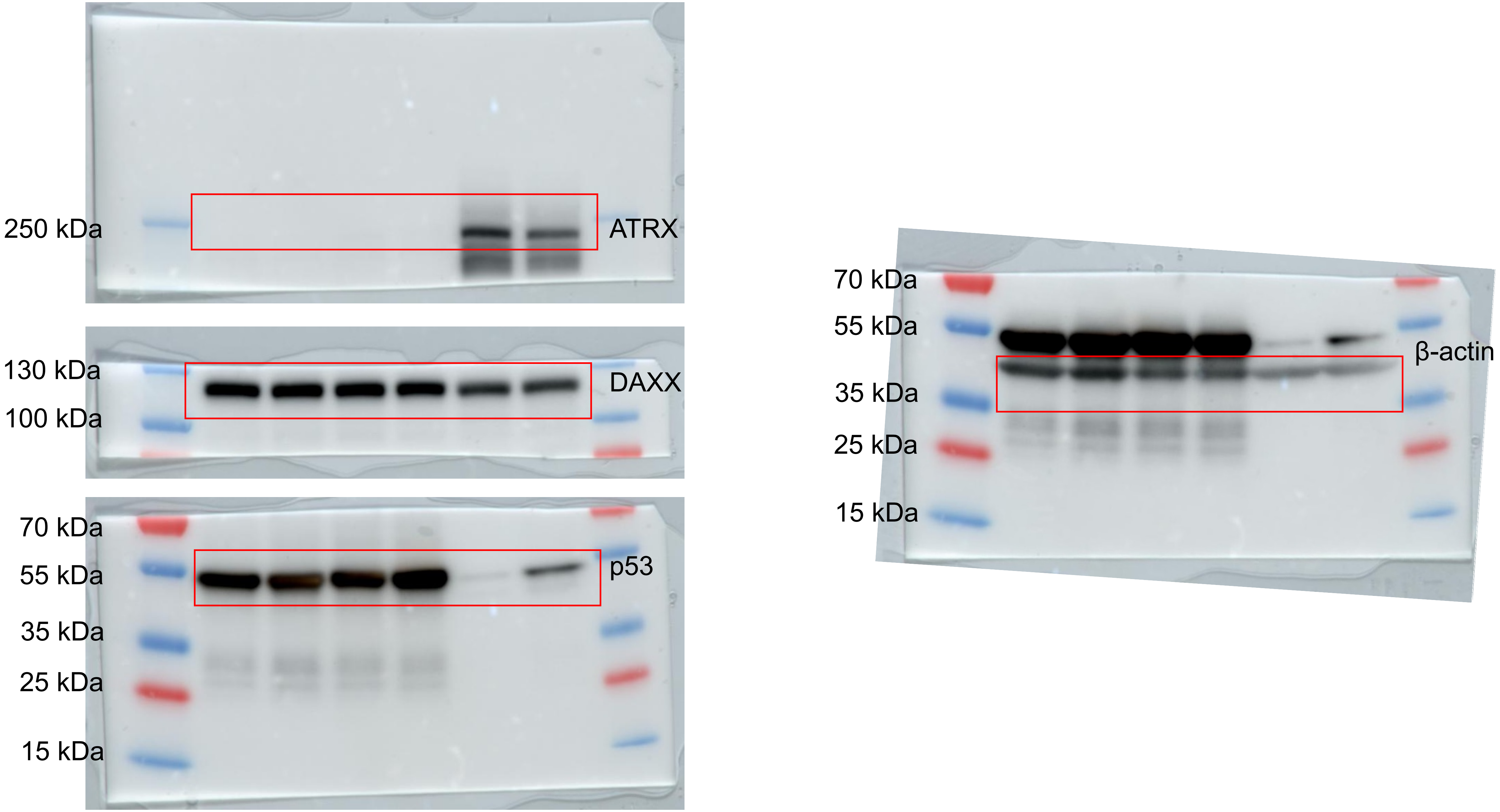

Supplementary Figure 9

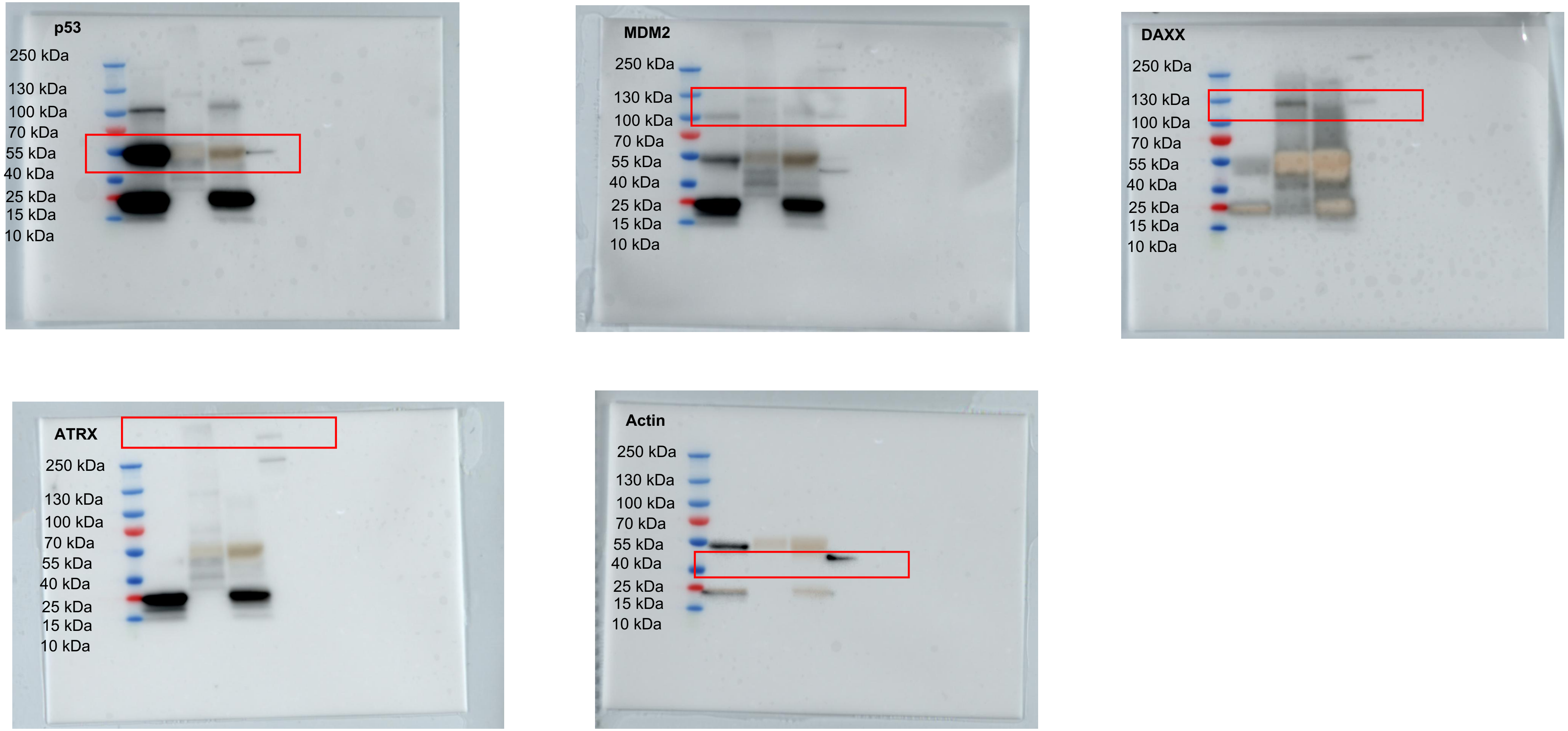

Supplementary Figure 10

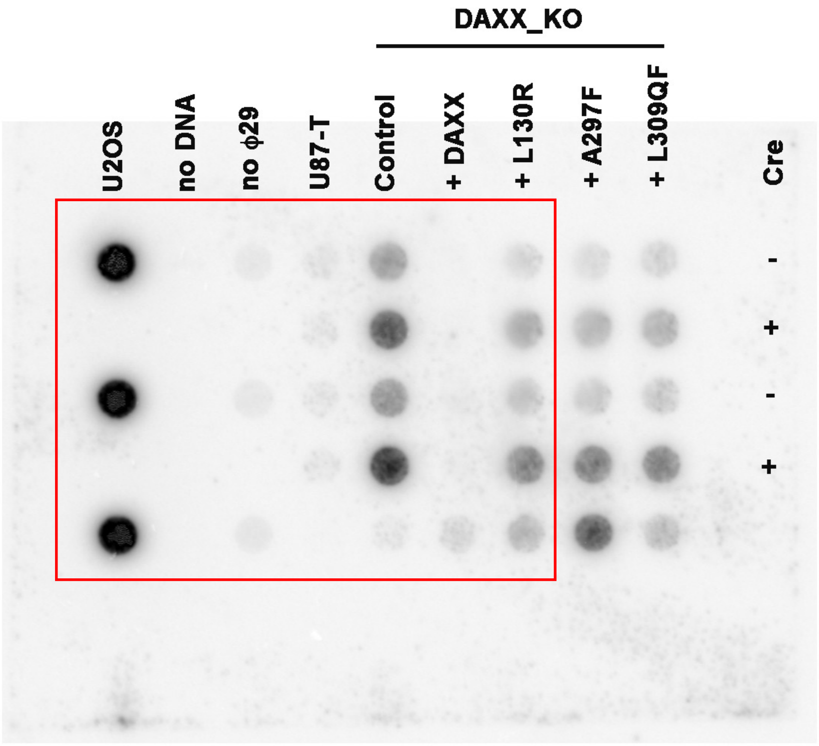

Cre is to remove hTERT from U87-T, row 3 is the image used in the figure

Supplemental Figure. Sequential Gating Strategy for Figure 2C, D, and E

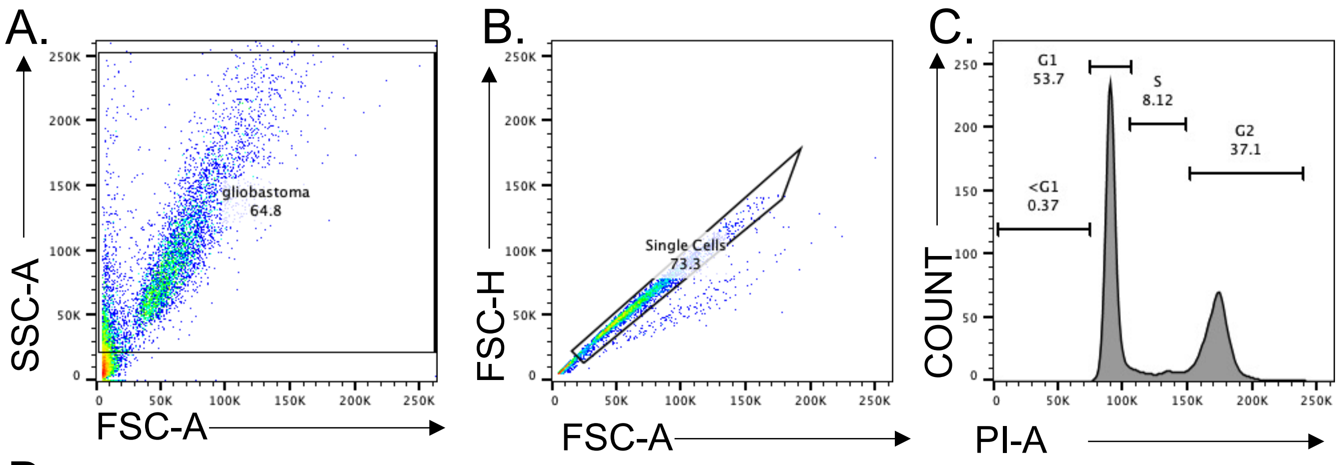

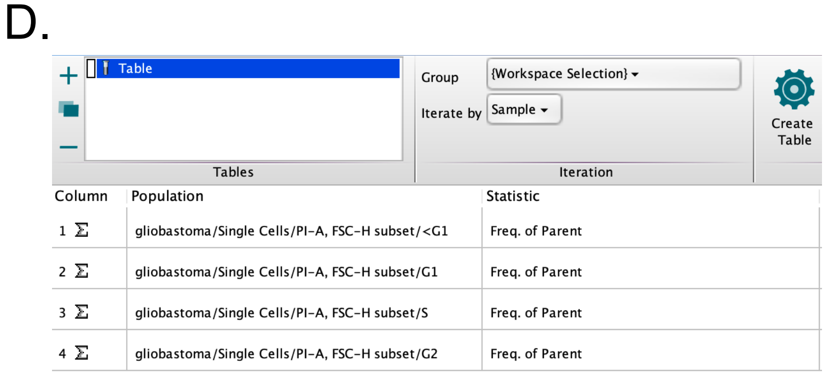

Supplemental Figure for Figure 2C, D, and E: **Gating Strategy** Triplicates for each condition were stained with propidium iodide (PI), as described. The DMSO controls were used to establish gating using FlowJo software A. First, we used forward scatter (FSC-A) and side scatter (SSC-A) to select the population to be analyzed. Rather than selecting only cells at the expected FSC-A and SSC-A population for healthy cells, we drew a wide gate encompassing all cells so that we could also account for cells undergoing apoptosis. B. Next, we performed doublet Doublet Discrimination using FSC-A and FSC-H to ensure that we counted only single cells and excluded doublets from our analysis. C. Again, using the DMSO treated control, we established gates for <G1 (apoptosis/necrosis), G1, S, and G2 by measuring PI-A and selected the "copy analysis to group" function to ensure that all gates (including total cell population, single cells, and <G1, G1, S, and G2) were applied uniformly to all samples. Representative cell cycle profiles for each condition are shown in **Figure 2D**. The table editor function was used to create a data table for all samples that included the population of "single cells" from each sample, giving the percentage for each sample that was gated in <G1, G, S, or G2. These results were exported from FlowJo into Excel to calculate the averages and standard deviations between triplicates of each sample. GraphPad Prism was then used to plot the percentage of each cell cycle phase observed in each condition (**Figure 2C**) and the percent change in S phase **Figure 2E**. Statistical analysis for **Figure 2E** was performed in GraphPad Prism.
